# YY1 is involved in homologous recombination inhibition at guanine quadruplex sites in human cells

**DOI:** 10.1093/nar/gkae502

**Published:** 2024-06-13

**Authors:** Xinyu Cui, Chengwen Zhang, Chunqing Fu, Jinglei Hu, Tengjiao Li, Lin Li

**Affiliations:** Shanghai Frontiers Science Center of Drug Target Identification and Delivery, School of Pharmaceutical Sciences, Shanghai Jiao Tong University, Shanghai 200240, China; National Key Laboratory of Innovative Immunotherapy, Shanghai Jiao Tong University, Shanghai 200240, China; Shanghai Frontiers Science Center of Drug Target Identification and Delivery, School of Pharmaceutical Sciences, Shanghai Jiao Tong University, Shanghai 200240, China; National Key Laboratory of Innovative Immunotherapy, Shanghai Jiao Tong University, Shanghai 200240, China; Shanghai Frontiers Science Center of Drug Target Identification and Delivery, School of Pharmaceutical Sciences, Shanghai Jiao Tong University, Shanghai 200240, China; National Key Laboratory of Innovative Immunotherapy, Shanghai Jiao Tong University, Shanghai 200240, China; Shanghai Frontiers Science Center of Drug Target Identification and Delivery, School of Pharmaceutical Sciences, Shanghai Jiao Tong University, Shanghai 200240, China; National Key Laboratory of Innovative Immunotherapy, Shanghai Jiao Tong University, Shanghai 200240, China; Shanghai Frontiers Science Center of Drug Target Identification and Delivery, School of Pharmaceutical Sciences, Shanghai Jiao Tong University, Shanghai 200240, China; National Key Laboratory of Innovative Immunotherapy, Shanghai Jiao Tong University, Shanghai 200240, China; Shanghai Frontiers Science Center of Drug Target Identification and Delivery, School of Pharmaceutical Sciences, Shanghai Jiao Tong University, Shanghai 200240, China; National Key Laboratory of Innovative Immunotherapy, Shanghai Jiao Tong University, Shanghai 200240, China

## Abstract

Homologous recombination (HR) is a key process for repairing DNA double strand breaks and for promoting genetic diversity. However, HR occurs unevenly across the genome, and certain genomic features can influence its activity. One such feature is the presence of guanine quadruplexes (G4s), stable secondary structures widely distributed throughout the genome. These G4s play essential roles in gene transcription and genome stability regulation. Especially, elevated G4 levels in cells deficient in the Bloom syndrome helicase (BLM) significantly enhance HR at G4 sites, potentially threatening genome stability. Here, we investigated the role of G4-binding protein Yin Yang-1 (YY1) in modulating HR at G4 sites in human cells. Our results show that YY1’s binding to G4 structures suppresses sister chromatid exchange after BLM knockdown, and YY1’s chromatin occupancy negatively correlates with the overall HR rate observed across the genome. By limiting RAD51 homolog 1 (RAD51) access, YY1 preferentially binds to essential genomic regions, shielding them from excessive HR. Our findings unveil a novel role of YY1–G4 interaction, revealing novel insights into cellular mechanisms involved in HR regulation.

## Graphical Abstract

**Graphical Abstract:**
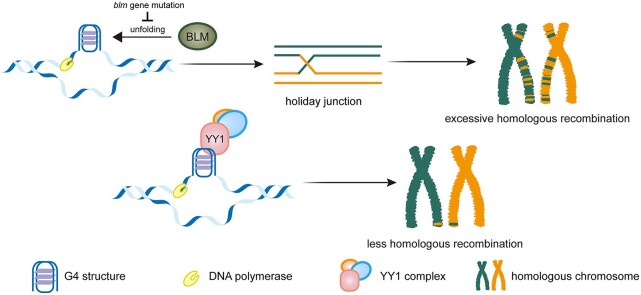

## Introduction

Homologous recombination (HR) is a fundamental biological process that is involved in DNA repair, chromosome segregation and genetic variation (1). In humans, HR occurs in both somatic and germ cells and is vital for genome stability modulation. The frequency of HR exhibits variance across the genome, featuring hotspots and cold spots. Several factors have been identified to modulate HR hotspots, among them PR/SET domain containing protein 9 (PRDM9) and ZCWPW1 (2–9). PRDM9 trimethylates H3K4 through its PR/SET domain at its binding sites, thereby activating homologous recombination (5–7). Concurrently, ZCWPW1, serving as a histone modification reader, recognizes H3K4me3 marks deposited by PRDM9 and promotes chromatin openness at recombination hotspots, subsequently promoting HR (10–12). Nevertheless, it is imperative to address the potential consequences of excessive or inadequate HR, which can lead to genome instability and the onset of various diseases, including cancer.

Guanine quadruplex (G4) structures are atypical secondary structures formed from DNA sequences with continuous repeats of guanines, known to form both *in vitro* and *in vivo* (13,14). Computational analysis of human genome has revealed an extensive array of over 376 000 motifs with the potential to form G4 structures (15), with a predominant localization observed at promoters or telomeres (16). Assisted by diverse G4-binding proteins, these structures undergo unwinding or stabilization, collectively shaping a dynamic G4 atlas (17). G4 formations exert substantial influence on cellular processes, including DNA replication, transcription, and repair (18). Furthermore, G4 structures have been implicated in inducing HR and DNA damage, attributed to their stable spatial arrangement that hinders DNA replication or transcription, consequently promoting genome instability and disease pathogenesis (4). Mutation of G4 helicases BLM or WRN results in genetic disorders such as Bloom's syndrome (BS) and Werner syndrome, respectively. (3,19,20). Individuals with these diseases typically exhibit developmental defects, premature aging, and increased risk of cancer (21,22). BLM is a mammalian RecQ family helicase that can bind to and unwind G4 structures *in vitro* and *in vivo* (18). Previous studies documented a direct association between G4 structure and HR in BLM-deficient cells, where HR preferentially occurs at G4 motifs in actively transcribed genes (23). However, our analysis revealed that the genome-wide recombination rates were lower at G4 structure loci than those without G4 structure, suggesting the presence of unknown mechanisms in protecting G4 structure sites from recombination.

Yin-Yang 1 (YY1) is a multifunctional zinc finger-containing transcription factor that is widely expressed and involved in various biological processes, including DNA replication, transcription, damage repair as well as cell proliferation, differentiation and embryogenesis (24). Recently, YY1 was identified as a novel G4-binding protein that regulates DNA looping and gene expression (25). Through cellular experiments and bioinformatic analysis, our study elucidates YY1’s capacity to modulate G4-mediated HR cold spots, thereby shielding essential and evolutionarily conserved genes from recombination.

## Materials and methods

### Cell culture

HEK293T cells were maintained in Dulbecco's modified Eagle's medium (DMEM, Thermo Fisher) supplemented with 10% fetal bovine serum (FBS, Adamas life) and 100 unit/ml penicillin/streptomycin at 37°C in a humidified incubator containing 5% CO_2_.

### Sister chromatid exchange (SCE) assay

SCE events were detected as described (26). Knockdown and overexpression were achieved by transfecting pSUPER plasmid carrying shRNAs targeting different genes (for knockdown) or pFL3 plasmid carrying YY1 sequence (for overexpression) (Supplementary Table S1) using Lipo293™ Transfection Reagent (Beyotime). After transfection for 48 h (knockdown and overexpression efficiency were shown in Supplementary Figure S1), HEK293T cells were cultured in a medium containing 5-bromo-2′-deoxyuridine (BrdU, AbMole Bioscience) about two cell cycles (40 h) to achieve sister chromatids labeling. All the operation after this step should be protected from light. To the culture medium was subsequently added colcemid (Rhawn) to a final concentration of 0.05 μg/ml to accumulate mitotic cells for 2 hr. The cells were then trypsinized, incubated with 60 mM KCl at room temperature for 20 min, fixed with a methanol-acetic acid (3:1, v/v) solution, and stored at 4°C. Prior to analysis, the cells were resuspended in a freshly prepared solution of methanol and acetic acid (3:1, v/v), spread onto a glass slide and air-dried at room temperature for 2 days. The slides were stained with 0.1 mg/ml acridine orange (Macklin) in water at room temperature for 5 min. After washing with Sorenson buffer (0.1 M Na_2_HPO_4_, 0.1 M NaH_2_PO_4_, pH 6.8), the slides were mounted and imaged using a Leica SP8 laser scanning microscope (Leica). The calculation was conducted by counting SCE numbers and chromosome numbers in a field of view, calculating SCE numbers/chromosome numbers. 10 images were obtained for each sample. *P* values were conducted by using two-tailed Student's *t*-test, presenting error bars using mean ± S.E.M. of results.

### Chromatin fractionation and western blot

Chromatin fractionation was performed as described (27). Briefly, the chromatin fraction was isolated using a step-wise procedure with a cytoplasmic lysis buffer (10 mM Tris–HCl, pH 8.0, 0.34 M sucrose, 3 mM CaCl_2_, 2 mM MgCl_2_, 0.1 mM EDTA, 1 mM DTT, 0.5% NP-40 and protease inhibitor cocktail), a nuclear lysis buffer (20 mM HEPES, pH 7.9, 1.5 mM MgCl_2_, 1 mM EDTA, 150 mM KCl, 0.1% NP-40, 1 mM DTT, 10% glycerol, protease inhibitor cocktail) and a chromatin isolation buffer (20 mM HEPES. pH 7.9, 1.5 mM MgCl_2_, 150 mM KCl, 10% glycerol, protease inhibitor cocktail and 0.15 unit/μl benzonase). The proteins were again quantified by Bradford assay.

Samples for western blot were lysed by cell lysis buffer (Beyotime, p2077589) and quantified by Bradford assay. After separation on a SDS-PAGE, the proteins were transferred to a 0.45μm PVDF membrane (Millipore). After blocking with blotting-grade blocker (Bio-Rad), the membrane was incubated in a solution containing primary antibody and 5% BSA for 2 h at room temperature, and then incubated in a 5% blotting-grade blocker containing HRP-conjugated secondary antibody. The western blot signal was detected using ECL western blotting detection reagent (Amersham). Primary antibodies used in this study included histone H3 (Cell Signaling Technology, 9715S; 1:10 000), RAD51 (Abcam, ab133534; 1:3000), GAPDH (Bioshare, SB-AB0038; 1:5000), BLM (Proteintech, 30254-1-AP, 1:1000), BRCA1 (Proteintech 22362-1-AP, 1:1000), SP1 (Abways, CY6610; 1:1000), NCL (Abways, CY7366; 1:1000) and YY1 (Santa Cruz, sc-7341; 1:200).

### Chromatin immunoprecipitation (ChIP)

ChIP experiments were conducted as previously described with a few modifications (25). Cells were cultured in DMEM medium with or without 20 μM PDS or 5 μM TMPyP4 for 12 h prior to cross-linking. For cells with knockdown treatment, cells were cultured in DMEM medium and transfected with pSUPER plasmid carrying shRNAs for 48 h prior to cross-linking. Approximately 2 × 10^6^ cells were cross-linked with 1% formaldehyde solution by rotating at room temperature for 10 min, and quenched with 125 mM glycine by rotating at room temperature for 5 min. After washing with cold PBS for 5 times, the cells were resuspended in 400 μl lysis buffer I (50 mM HEPES–KOH, pH 7.5, 140 mM NaCl, 1 mM EDTA, 10% glycerol, 0.5% NP-40, 0.25% Triton X-100, protease inhibitors cocktail) at 4°C on a rotator for 10 min. After centrifugation at 10 000 RPM at 4°C for 5 min, the pellet was resuspended in 250 μl lysis buffer II (10 mM Tris–HCl, pH 8.0, 200 mM NaCl, 1 mM EDTA, 0.5 mM EGTA, protease inhibitor cocktail) at 4°C for 10 min with rotation. After the same centrifugation condition before, the pellet was resuspended in 200 μl sonication buffer (20 mM Tris–HCl, pH 8.0, 150 mM NaCl, 2 mM EDTA, 0.1% SDS, 1% Triton X-100 and protease inhibitor cocktail). 200 μl solution in a 1.5 ml tube was used for the sonication. Sonication was conducted using a ultrasonicator (ScientZ, JY92-II N) with a 3 mm tip at 4°C, with 3% power output, 5 s on and 10 s off for total 8 min. The shearing size of the chromatin was usually 200–500 bp. The supernatant was incubated with anti-RAD51 (Abcam, ab133534), anti-YY1 (Santa Cruz, sc-7341) or anti-H3K4me3 (Abcam, Ab8580) antibody at 4°C overnight (800 ng chromatin using 1 μg antibody). To the mixture was added protein A + G Plus-Agarose (Beyotime, P2055), and the mixture was incubated at 4°C for 4 h. After washing with cold PBS for 5 times, DNA was subsequently eluted from the beads with 100 mM NaHCO_3_ and 1% SDS at 68°C for 2 h. Cross-links were subsequently reversed by incubating at 65°C overnight and RNA was removed with RNase A. For BG4 ChIP, the chromatin was incubated with BG4 antibody at 16°C for 2 h (800 ng chromatin using 500 ng antibody). To the mixture was added anti-Flag COIP beads (Bioshare, SB-PR002), and the mixture was incubated at 16°C for 1 h. After washing with washing buffer (10 mM Tris–HCl, pH 8.0, 0.1% Tween-20, 100 mM KCl) for 5 times, DNA was eluted from the beads with TE buffer at 37°C for 1 h. Cross-links were subsequently reversed by incubating at 65°C for 2 h and RNA was removed with RNase A. Finally, the DNA was purified using UNIQ-10 Column MicroDNA Gel Extraction Kit (Sangon, B511139-0050).

### Real-time quantitative PCR (RT-qPCR)

RT-qPCR was conducted as previously described (27). Total RNA was extracted using Promega Total RNA Kit (Promega, LS1040) and quantified. Reverse transcription was performed using Reverse Transcriptase (Bioshare, SB-RT001) for cDNA synthesis. RT-qPCR was carried out using 2*Universal SYBR Green qPCR Premix (Vazyme, Q312-02) on a RT-qPCR detection system (StepOne PLUS), following the manufacturer's recommended procedures. Primers used for RT-qPCR were listed in Supplementary Table S2.

### BG4 antibody expression and purification

BG4 expression and purification were performed as described previously (28) with minor modifications: *Escherichia coli* BL21 Star (DE3) were transformed with pSANG10-3F-BG4 (Addgene #55756). Cells were resuspended in IMAC buffer lysis buffer (100 mM HEPES pH 8.0, 500 mM NaCl, 10% glycerol, 10 mM imidazole, 1 mM PMSF) and homogenized using 30 kPSI pressure. The lysate was centrifuged twice (25 min, 14 000 RPM at 4°C), the supernatant was mixed with 1 ml 50% His-tag Purification Resin (Beyotime, P2233) and were loaded on an Empty Affinity Chromatography (AC) Column Kits (Beyotime, FCL03). The column was washed with IMAC wash 1 buffer (20 mM HEPES pH 7.5, 500 mM NaCl, 10% glycerol, 10 mM imidazole), IMAC wash 2 buffer (20 mM HEPES pH 7.5, 500 mM NaCl, 10% glycerol, 50 mM imidazole) and eluted with IMAC elution buffer (20 mM HEPES pH 7.5, 500 mM NaCl, 10% glycerol, 500 mM imidazole). The concentration of FLAG-tagged BG4 was determined with Coomassie brilliant blue staining (Supplementary Figure S2).

### pA-Tn5 expression and purification

The 3 × FLAG sequences in 3 × Flag-pA-Tn5-Fl (Addgene #124601) were replaced with 10 × His sequence. MXE GyrA intein sequence was deleted. *Escherichia coli* Rosetta (DE3) was transformed with 10 × His-pA-Tn5 and pre-cultured overnight at 37°C in 100 μg/ml Ampicillin. 200 ml LB, 100 μg/ml Ampicillin was inoculated with 2 ml of the overnight culture and grown at 37°C until OD 0.6. IPTG was added to 1 mM and expression was carried out at 23°C for 5 h. Cells were pelleted and washed with PBS buffer by agitation at 4°C. The purification protocol is same as the BG4 purification. Lysis buffer is HXG buffer (20 mM HEPES-KOH pH 7.2, 800 mM NaCl, 10% glycerol, 0.2% Triton X-100). The wash buffer is HXG buffer with 10 mM imidazole and the elution buffer is HXG buffer with 250 mM imidazole. Poly(ethyleneimine) was used to remove the pA-Tn5 that bound with DNA. pA-Tn5 was dialyzed using 2 × Tn5 dialysate (100 mM HEPES–KOH (pH 7.2), 200 mM NaCl, 0.2 mM EDTA, 2 mM DTT, 0.2% Triton x-100, 20% glycerol) and then mixed with 1 × volume glycerol. The concentration of Tn5 was determined with Coomassie brilliant blue staining (Supplementary Figure S2). pA-Tn5 was assembled with Tn5MEDS-A and Tn5MEDS-B as previously described (29,30).

### Cleavage under targets and tagmentation (CUT&Tag)

CUT&Tag experiments were performed as described previously (28–30) with minor modifications: Briefly, 2 × 10^5^ cells were treated with NE1 buffer (20 mM HEPES–KOH pH 7.9, 10 mM KCl, 0.5 mM Spermidine, 0.1% (v/v) Triton X-100, 20% (v/v) Glycerol) on ice for 10 min, then fixed with 0.1% formaldehyde in PBS for 2 min and harvested. The cells were washed with PBS, and immobilized to Concanavalin A Magnetic Beads (Beyotime, P2156) with incubation at room temperature for 10 min. The bead-bound cells were incubated in 200 μl of primary antibody buffer (20 mM HEPES–KOH pH 7.5, 150 mM NaCl, 0.5 mM Spermidine, 1% (v/v) BSA, 2 mM EDTA) with 40 μl 0.05 mg/ml FLAG-tagged BG4 antibody or 2 μl 1 mg/ml Mouse IgG Isotype Control (Share-bio, SB-A00001) antibody at 4°C by rotating overnight. The next day, Flag-Tag Mouse Monoclonal Antibody (Share-bio, SB-AB0008), Rabbit Anti-Mouse IgG H&L antibody (Bioss, bs-0296R), pA–Tn5 adapter complex were incubated at 37°C for 1 h. All three antibodies were diluted as 1:200. Flag-Tag Mouse Monoclonal Antibody and Rabbit Anti-Mouse IgG H&L antibody were diluted with BSA-wash buffer (20 mM HEPES–KOH pH 7.5, 150 mM NaCl, 0.5 mM Spermidine, 1% (v/v) BSA), and pA-Tn5 adapter complex was diluted with BSA-300 buffer (20 mM HEPES–KOH pH 7.5, 300 mM NaCl, 0.5 mM spermidine, 1% (v/v) BSA). After each incubation of antibodies, the beads were washed 3 times with the corresponding antibody incubation solution for 5 min. Tagmentation was performed with 50 μl tagmentation buffer in 37°C for 1 h (20 mM HEPES–KOH pH 7.5, 300 mM NaCl, 0.5 mM spermidine, 1% (v/v) BSA, 10 mM MgCl_2_). After tagmentation, 1 μl 500 mM EDTA, 1 μl 20 mg/ml proteinase K and 1 μl 10% (w/v) SDS were added and further incubated in 55°C for 1 h and 70°C for 20 min to stop tagmentation and digest protein. 122 μl DNA Selection Beads (Yeason, 12601ES) were added to the supernatant of the aforementioned process. Wash twice in 80% ethanol and elute the DNA using 35 μl TE buffer. To generate G4 libraries, purified genomic DNA was amplified with the universal i5 primer and barcoded i7 primer using NEBNext Ultra II Q5 Master Mix (NEB, M0544). The library PCR products were electrophoresed using 2% (w/v) agarose gel and 200–500 bp size fragments were recovered with Gel Extraction Kit (Sangon Biotech, B511139). Sequencing was conducted on an Illumina Novaseq-PE150 instrument.

For CUT&Tag data analysis, after quality checks with FastQC, the reads were aligned to human hg19 reference genome using Bowtie2 (v.2.5.0) tool with the configuration of bowtie2 -q -N 0 -L 22 (31). Peak calling was performed using the model-based analysis of ChIP–seq (MACS2 v.2.2.9.1) with the following configuration: MACS2 callpeak -f BAM -g 2.7e + 9 –n BG4 CUT&Tag (32). Integrative genomics viewer (IGV) was used to visualize the CUT&Tag signal (33).

### Immunofluorescence microscopy

BG4 and RAD51 immunofluorescence experiments were performed essentially as described (34) with slight modifications. In brief, 1 × 10^5^ HEK293T cells were seeded overnight on circular coverslips per well in 24-well plates. Next day, cells were transformed with plasmids for 48 h. Cells were fixed with 4% (v/v) freshly prepared paraformaldehyde in 1 × PBS for 10 min at room temperature. Coverslips were permeabilized with 0.1% (v/v) Triton-X100 in PBS for 10 min, and blocked with 2% (w/v) Skim milk (Beyotime, P0216) in PBS. After blocking, coverslips were incubated with BG4 (1 nM) at 37°C for 1 h, and then incubated with Flag-Tag Mouse Monoclonal Antibody (Share-bio, AB0008; 1:1000) and Rabbit Anti-Rad51 Antibody (abcam, ab133534, 1:1000). Subsequently, all coverslips were incubated 1 h at 37°C with Goat Anti-Rabbit IgG(H + L) Alexa Fluor 594 (Share-bio, SB-AB0151, 1:1000) and Goat Anti-Mouse IgG (H + L) Alexa Fluor 488 (Share-bio, SB-AB0142, 1:1000). DAPI nuclear stain was performed after the final antibody incubation. Coverslips were mounted onto Adhesion Microscope slides (Titan, 02036395) with Antifade Mounting Medium (Beyotime, P0126). Three biological replicates were performed. Images were acquired on a Leica Stellaris 5 Cryo Confocal Light Microscope. Fluorescence illumination was with a xenon source and Alexa 488, Alexa 594, and DAPI fluorescent filter cubes used to image BG4, RAD51, and the nuclei, respectively. Images were processed using LAS X 3.7.4 Software (Leica). The quantification was processed using ImageJ. The integrated fluorescence intensity values were background subtracted and normalized to the nuclear area. The overlap analysis was conducted using MatCol (35) with default parameter.

### Electrophoretic mobility-shift assay (EMSA)

The RAD51 protein (Elabscience, PDEH100341) and BG4 protein was stained by Coomassie brilliant blue for quality verification (Supplementary Figure S2). For G4 structure formation, c-KIT and c-MYC were annealed by incubating in 100°C for 5 min followed by slow cooling to room temperature in an annealing buffer containing 10 mM Tris–HCl (pH 7.5), 100 mM KCl and 0.1 mM EDTA. The ss-DNA was dissolved in TE buffer. For protein-DNA binding, 10 nM DNA (Supplementary Table S3) was incubated with different concentrations of full-length RAD51 in a binding buffer containing 20 mM Tris–HCl (pH 8.0), 30 mM NaCl, 1 mM DTT, 2 mM ATP, 5 mM CaCl_2_ and 0.2% Tween 20 at 37°C for 15 min, or BG4 in a binding buffer containing 20 mM Tris–HCl (pH 8.0), 30 mM KCl, 1 mM MgCl_2_, 1 mM DTT and 0.2% Tween 20 at room temperature for 30 min. Glycerol was added before loading to 5% as the final concentration. The samples were than loaded onto a 4% polyacrylamide gel in TBE buffer (45 mM Tris, 1 mM EDTA·2Na, 45 mM boric acid) at 4°C. The samples were run at 120 V at 4°C for 20 min. For not TAMRA-labelled DNA, SYBR Gold (Invitrogen) was used for gel staining. The gels were imaged with Bio-Rad CFX Opus 96.

### CD spectroscopy

CD spectroscopy was performed as described previously (25). In brief, the DNA probes (2.5 μM) were annealed in buffer containing 10 mM Tris–HCl (pH 7.5), 100 mM KCl and 0.1 mM EDTA to form G4 structure. The CD spectra for the DNA probes were recorded at room temperature on a Jasco-815 spectrometer in the wavelength range of 200–320 nm, and the scan rate was 1 nm s^−1^.

### Bioinformatic analysis

Average genetic recombination rate was calculated by averaging matched recombination rate in genetic map of the center of each ChIP-seq peaks. The genetic map was from the 1000 Genomes Project (36) and the custom code for calculation was provided in data availability. The overlap between ChIP-seq datasets was analyzed using bedtools (v.2.30.0) with the configuration of -a inputfile1.bed -b inputfile2.bed -wa (37). ChIP-seq signal tracks were visualized using IGV (33). Deeptools2 (v.3.5.4) (38) computeMatrix was employed to calculate the signal of each 50 bp bin in the regions of the peak center ±5 kb. The aggregation plot was generated using Deeptools2 plotProfile with default configuration. The average recombination rates around ChIP-seq peaks ±5 kb were calculated by custom code using recombination rate in genetic map from the 1000 Genomes Project. In detail, recombination rate of every center position of each peak and ±5 kb were averaged respectively. The related figures were plotted by R (v.4.3.0) using custom code. Average minor allele frequency (MAF) of specific ChIP-seq data was calculated by averaging matched MAFs of all positions of ChIP-seq peaks from common dbSNP database using custom code (39). CRISPR scores for YY1 and PRDM9 were obtained from CRISPR score database (40). The Gene Oncology (GO) bubble chart was plotted by R. The ChIP-seq datasets annotation pie charts were analyzed and plotted by ChIPseeker (41). The G4 hunter frequency was obtained from http://bioinformatics.ibp.cz. (42). Details of codes for several calculation are in code availability.

## Results

### Screening for G4-binding proteins that modulate homologous recombination

**Figure 1.**
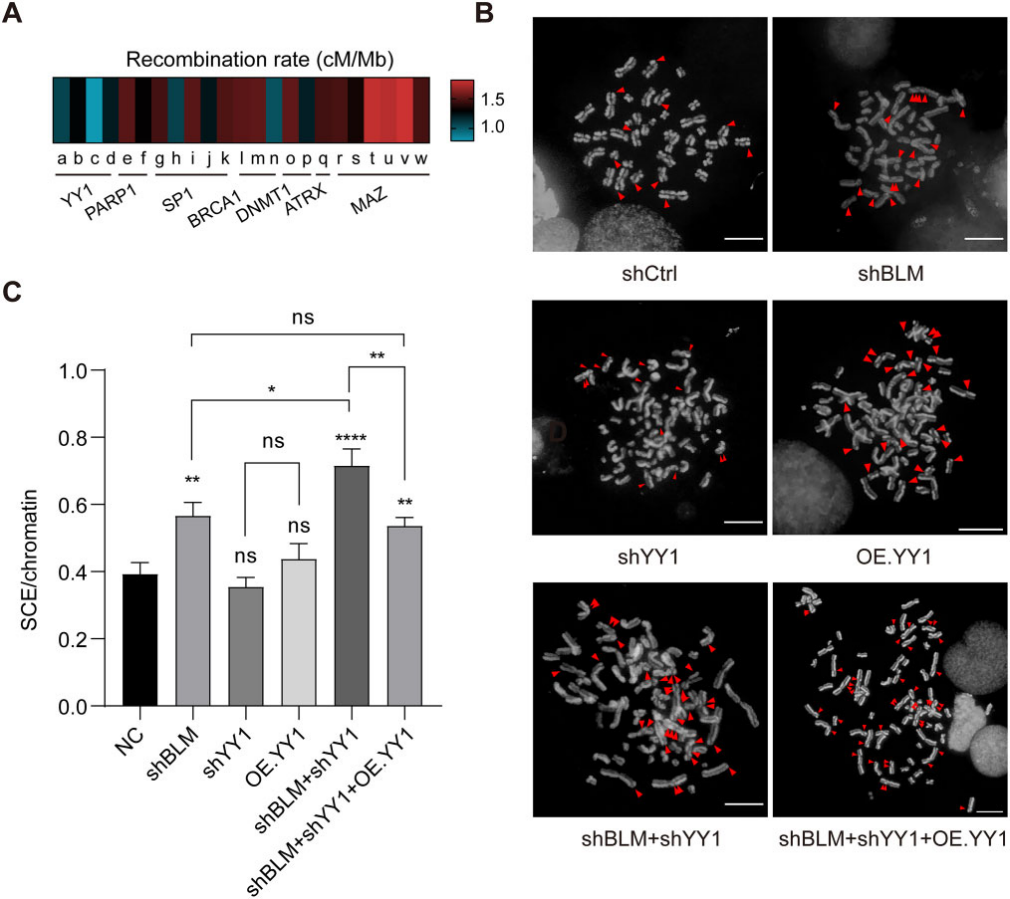
YY1 suppresses BLM deficiency-mediated HR. (**A**) A heat map depicting average recombination rate from ChIP-seq datasets of screened G4-binding proteins of different cell lines. The accession numbers for each dataset used and their corresponding cell line information are listed in Supplementary Table S4 (*n* > 900). (**B**) SCEs are shown in control HEK293T or after treatment with shBLM, shYY1, OE.YY1, shBLM + shYY1 or shBLM + shYY1 + OE.YY1. SCEs were marked with red arrowhead. Scale bar = 30 μm. (**C**) Quantification of SCEs per chromatin in control HEK293T or after treatment with shBLM, shYY1, OE.YY1, shBLM + shYY1 or shBLM + shYY1 + OE.YY1 (*n* > 200). The *P* values were calculated by using two-tailed Student's *t*-test: *P < 0.05; **P < 0.01; ****P < 0.0001. The data represent mean ± S.E.M. of results.

It remains unknown whether there exists cellular mechanism(s) suppressing HR at G4 sites. We initiated our investigation by probing whether G4-binding proteins regulate HR at G4 structure loci. To this end, we selected a group of G4-binding proteins that are mainly involved in DNA metabolism and damage repair from G4 Interacting Proteins DataBase (G4IPDB). We then computed the average recombination rate of their binding sites across the genome utilizing data from the 1000 Genomes Project (36,43) (Figure 1A and Supplementary Table S4). We found that the binding sites of several G4-binding proteins, including YY1, poly (ADP-ribose) polymerase 1 (PARP1), transcription factor Sp1 (SP1), and breast cancer type 1 susceptibility protein (BRCA1), exhibited relatively low recombination rates compared to DNA (cytosine-5)-methyltransferase 1 (DNMT1), transcriptional regulator ATRX, and myc-associated zinc finger protein (MAZ). Particularly noteworthy was the observation that the binding sites of the G4-binding protein YY1 exhibited the lowest recombination rate in our dataset (Figure 1A). Of significance, PARP1, SP1 and BRCA1 represent pivotal DNA damage repair proteins, in addition to their roles as G4-binding proteins, which may also impact HR processes within cells (44–46). Moreover, G4-binding protein nucleolin (NCL) was shown to facilitate cellular responses to DNA damage and was associated with the HR process (47,48). Hence, we embarked on exploring the potential roles of these proteins in G4-mediated HR.

### YY1 suppresses G4 structure-mediated HR

Sister chromatid exchange events (SCEs) are hallmark of genomic instability and byproduct of DNA damage repaired by HR in Bloom's syndrome cells (21,23,49,50). Deficiency in BLM leads to increased G4 formation and elevated SCEs, rendering it a suitable model for studying G4-mediated HR (51). Hence, we assessed SCEs in HEK293T cells after shRNA-mediated knockdown of BLM and selected G4-binding proteins, either individually or in combination, using SCE assay. This assay utilizes differential staining of sister chromatids, thereby enabling the microscopic detection of DNA exchange events occurring through homologous recombination (HR). It stands as the most established method for identifying dysregulated HR processes (52). A single SCE is defined as sister chromatids exchanging with each other once (Supplementary Figure S3A).

We found that single knockdown of PARP1, BRCA1 or NCL led to augmented SCEs in HEK293T cells, while knockdown of them together with BLM showed minor increased SCEs in cells, primarily attributable to the effect of BLM itself (Supplementary Figure S3B and S3D), consistent with their roles in DNA damage repair (47,48,53,54). Based on these results, we hypothesize that the effects of PARP1/BRCA1/NCL and BLM are merely additive, and there is no synergistic enhancement of G4-related SCE induced by BLM depletion by PARP1/BRCA1/NCL. Knockdown of another G4 binding protein, SP1, did not yield statistically significant impacts on SCE events (Supplementary Figure S3B and S3D). Neither knockdown nor overexpression of YY1 alone appreciably affected SCEs; however, concurrent knockdown of YY1 with BLM resulted in a significantly higher number of SCEs compared to knockdown of BLM alone (Figure 1B, C). Similar results were observed in the NCI-H125 cell line (Supplementary Figure S3C and S3E). Moreover, when YY1 was overexpressed in shBLM + shYY1 cells to rescue YY1 expression, the level of SCEs resembled that of BLM-deficient cells (Figure 1B, C). We next sought to determine whether the elevation of SCEs induced by YY1 knockdown might be attributed to changes in G4 levels. Immunofluorescence imaging with a G4-specific antibody BG4 was utilized to assess cellular G4 levels following YY1 modulation. Our results revealed that neither overexpression nor knockdown of YY1 significantly altered overall intracellular G4 levels (Supplementary Figure S4), indicating that YY1 does not directly affect G4 formation. These observations introduce a potential role of YY1 in suppressing G4-mediated HR.

**Figure 2.**
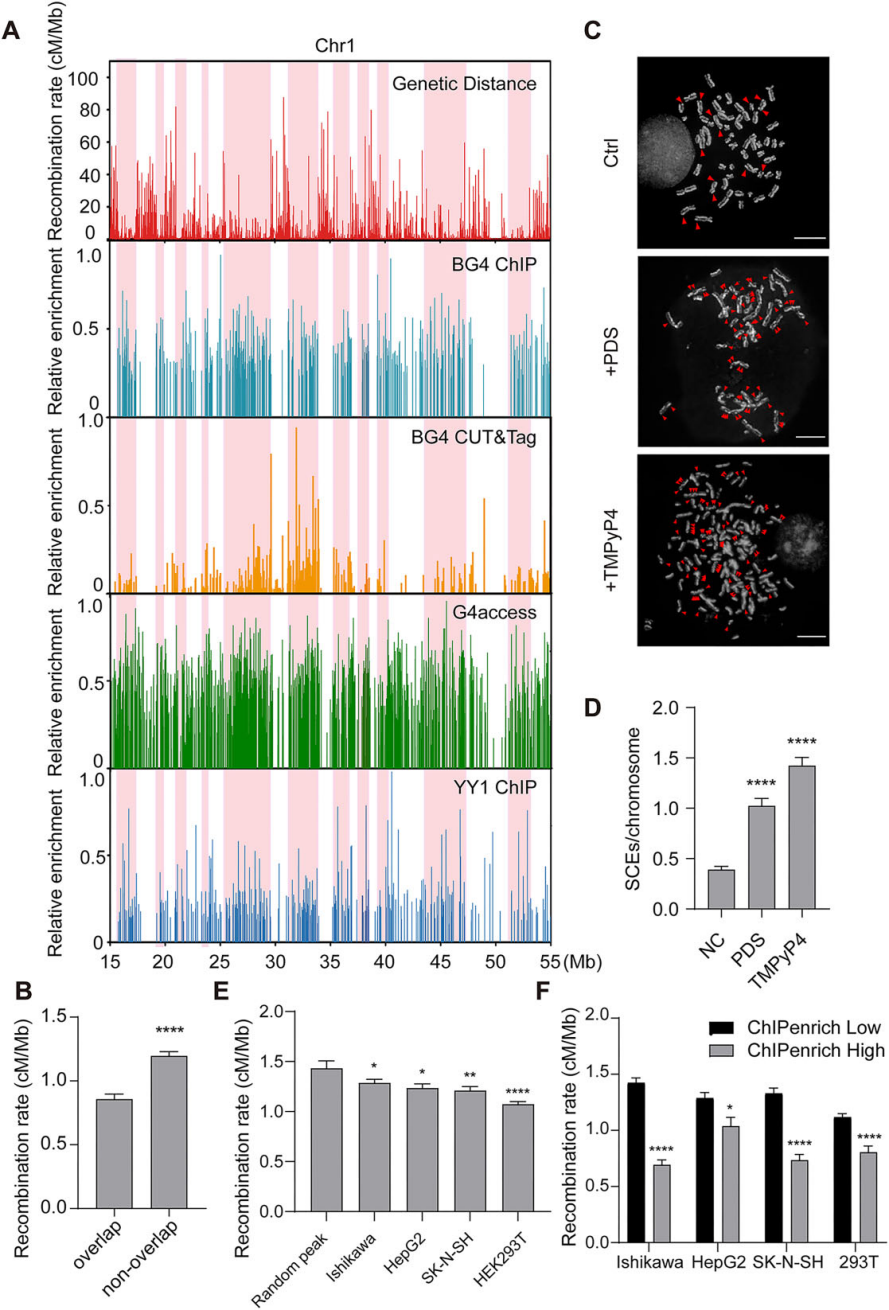
YY1 functions as a suppressor for G4 structure-mediated HR. (**A**) Comparison of genetic map and ChIP-seq peaks from a region on chromosome 1 (15–55 Mb). Four datasets were used including BG4 ChIP-seq (second panel), BG4 CUT&Tag (third panel), G4access (fourth panel) and YY1 ChIP-seq (bottom panel). Regions with low recombination rates but high ChIP-seq enrichment were marked in pink columns. (**B**) A comparison of average recombination rates of G4 sites overlap or non-overlap with YY1 binding (*n* > 3500). (**C**) SCEs in 293T cells with or without treatment with PDS or TMPyP4. SCEs were marked with red arrowhead. Scale bar = 30 μm. (**D**) Quantification of SCEs per chromatin with or without treatment with PDS or TMPyP4 (*n* > 200). (**E**) A comparison of genetic distance between the region 10 kb upstream and 10 kb downstream of the random peaks or YY1 ChIP-seq peaks from four datasets (*n* > 8000). (**F**) A comparison of genetic distance between ChIP-seq peaks with low (relative enrichment lower than 0.2) and high (relative enrichment higher than 0.2) enrichment of YY1 occupancy (*n* > 1800). *P* values were calculated by using two-tailed Student's *t*-test: *P < 0.05; **P < 0.01; ****P < 0.0001. The data represent mean ± S.E.M. of results.

We proceeded to investigate whether YY1 regulates HR universally. Since YY1 is a G4-binding protein, we compared the distribution of YY1 ChIP-seq peaks and G4 ChIP-seq peaks with the genetic map of recombination rates across the human genome (25,36). We found that YY1 ChIP-seq peaks were enriched in genomic regions with low recombination rates, especially those regions with high enrichment folds and overlapping with G4 ChIP-seq peaks (Figure 2A and Supplementary Figure S5A–S5D). Furthermore, by calculating the genome-wide average recombination rates of G4 sites overlapping with YY1 binding or not, we observed that YY1-bound G4 sites exhibited lower recombination rates (Figure 2B), with over 80% of BG4 and YY1 overlapping peaks enriching in regions where recombination rates were below average (Supplementary Figure S5E), suggesting that YY1 suppresses HR at G4 structure sites. Moreover, the number of SCEs increased dramatically by over two times upon treatment with G4-binding ligands, i.e. pyridostatin (PDS) or 5,10,15,20-tetra-(*N*-methyl-4-pyridyl) porphyrin (TMPyP4) (Figure 2C, D), which were already proved to compete with YY1 in binding with G4 (Supplementary Figure S5F) (25,55,56).

We further explored the role of YY1 in modulating HR by assessing the correlation between recombination hotspots in the human genome and chromatin occupancy of YY1 from four ChIP-seq datasets acquired for different cell lines (Ishikawa, HepG2, SK-N-SH and HEK293T). We compared these datasets with the genetic map of recombination hotspots, and the results showed that YY1-occupied genomic regions exhibited significantly lower recombination rates than random regions (Figure 2E). Moreover, regions enriched with YY1 displayed diminished recombination rates (Figure 2F). Together, these results indicate that deficiency in BLM leads to increased G4 formation and elevated HR at these sites, whereas binding of YY1 to G4 structures suppresses these sites from participating in HR.

### YY1 suppresses HR by impeding the chromatin localization of RAD51

**Figure 3.**
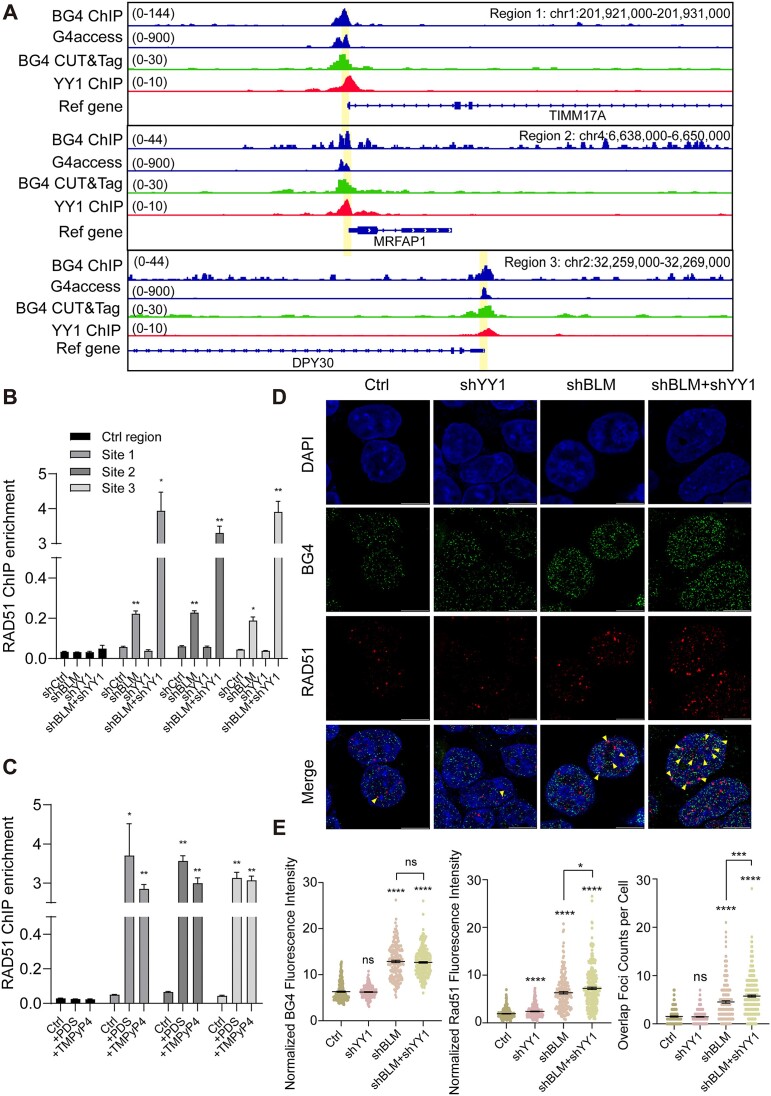
YY1 suppresses HR by repressing chromatin localization of RAD51 at genome-wide scale. (**A**) ChIP-seq signals of G4 sites 1–3 in B and C. The amplified regions of ChIP-qPCR in B and C were in yellow shadow. (**B**) ChIP-qPCR enrichment of RAD51 after treatment with shBLM, shYY1 or shBLM + shYY1 in control region and G4 sites 1–3 in HEK293T (*n* = 3). (**C**) ChIP-qPCR enrichment of RAD51 after treatment with or without PDS and TMPyP4 in control region and G4 sites 1–3 in HEK293T (*n* = 3). (**D**) Representative fields of view showing G4 and RAD51 foci formation detected by immunofluorescence in control (Ctrl), shYY1, shBLM and shBLM + shYY1 treated HEK293T cells. Nuclear staining (blue), BG4 (green), RAD51 (red) and the merged channels are reported. The colocalization of RAD51 and BG4 were pointed with yellow arrowheads. (**E**) Quantification of BG4 (left) and RAD51 (middle) nuclear staining detected by immunofluorescence in control (Ctrl), shYY1, shBLM and shBLM + shYY1 HEK293T cells. Quantification of the overlap foci counts per cell detected by immunofluorescence in control (Ctrl), shYY1, shBLM and shBLM + shYY1 HEK293T cells (right). Scale bars = 10 μm. *P* values were calculated by using two-tailed Student's *t*-test: *P < 0.05; ***P < 0.001; ****P< 0.0001. All data represent mean ± S.E.M. of results.

DNA repair protein RAD51 homolog 1 (RAD51) is a key recombinase involved in DNA double-strand break repair by locating and invading homologous DNA sequences to promote HR (57). Firstly, we verified the binding activity of RAD51 with a single-stranded DNA (ssDNA) probe (Supplementary Figure S6C). The formation of G4 structures used in the EMSA assay was confirmed through circular dichroism (CD) spectroscopy (Supplementary Figure S6C-S6G). EMSA assay revealed that RAD51 is not a G4 binding protein, excluding its function through directly binding with G4 (Supplementary Figure S6A and S6B). We next conducted BG4 CUT&Tag sequencing in HEK293T cells. The results showed good consistency with two public databases (labelled as BG4 ChIP-seq and G4access in Fiuge 3A). In order to choose proper G4 sites to investigate the occupancy of YY1 and RAD51, we compared the BG4 CUT&Tag sequencing together with YY1 ChIP-seq data in HEK293T cells. Three G4-enriched genomic sites were used based on the criteria that it could form G4 and YY1 could bind there (Figure 3A). We further confirmed the G4 formation at these sites by G4 hunter tool (a tool for G4 forming prediction), showing potential G4 formation in three G4-enriched sites with the frequency 12, 8 and 6 compared to 0 of control region (Supplementary Figure S6H and Supplementary Table S5) (42). Next, we investigated the G4 levels at those three G4-enriched sites after TMPyP4 or PDS treatment by BG4 ChIP-qPCR, further confirming the G4 formation (Supplementary Figure S6I–S6J).

We next wanted to elucidate whether elevated G4 formation by BLM deficiency could promote RAD51’s occupancy. We confirmed that BLM knockdown gave rise to increased enrichment in RAD51 ChIP signal at three G4-enriched sites relative to the control region (Figure 3B and Supplementary Figure S7A and S7B). And this elevation of RAD51 enrichment was also observed in four other well-established G4-enriched gene regions (Supplementary Figure S7C). In our proposed model, where YY1 acts as a repressor for HR at G4 sites, RAD51’s occupancy at G4 sites should elevated after YY1 depletion. When comparing the RAD51’s ChIP enrichment after knockdown of BLM, YY1 or both, we observed statistically significant increase in RAD51 enrichment at these three G4 sites, while YY1 alone did not affect RAD51’s ChIP enrichment, indicating that YY1 suppressed the recruitment of RAD51 to G4 loci in BLM-deficient cells and thereby prevented HR in these regions (Figure 3B). Importantly, knockdown of YY1 and BLM together resulted in a significant increase in chromatin-bound of RAD51, while knockdown of YY1, BLM or both did not influence the total cellular expression of RAD51 (Supplementary Figure S7B). In addition, to ensure that RAD51 enrichment is specific to G4 sites, RAD51 ChIP-qPCR at non-G-rich regions of high recombination rate on Chr1 to Chr4 was performed. Results revealed that the RAD51 signal enhancement after knockdown of BLM could not be observed at non-G-rich regions (Supplementary Figure S7D).

PDS and TMPyP4 are G4 stabilizers that compete with YY1’s binding to G4 (25). Treatment with PDS or TMPyP4 could elevate cellular G4 levels and suppress YY1’s binding at G4 sites (25). Therefore, if YY1 indeed suppresses RAD51 recruitment at G4 sites, treatment with PDS or TMPyP4 should significantly increase RAD51 signal and HR events at G4 sites. Indeed, SCEs increased prominently after PDS or TMPyP4 treatment (Figure 2C), and ChIP-qPCR results exhibited a significant augmentation in RAD51 enrichment at G4 sites after treatment, aligning with our proposed model (Figure 3C and Supplementary Figure S7E–S7F).

To provide further support for our hypothesis, we conducted immunofluorescence staining of G4 and RAD51 in HEK293T cell following knockdown of YY1, BLM or both (Figure 3D). Consistent with our proposed model, simultaneous knockdown of BLM and YY1 resulted in more than a twofold increase in both G4 and RAD51 intensity, particularly with a significant increase in the co-localization of G4 and RAD51 (Figure 3D and E). In contrast, knockdown of YY1 alone did not affect the colocalization of G4 and RAD51 (Figure 3D and E). These data suggest that at G4 sites, YY1 binding suppresses recruitment of RAD51 and thereby prevents HR.

### YY1 suppresses HR at genomic loci enriched with essential genes

**Figure 4.**
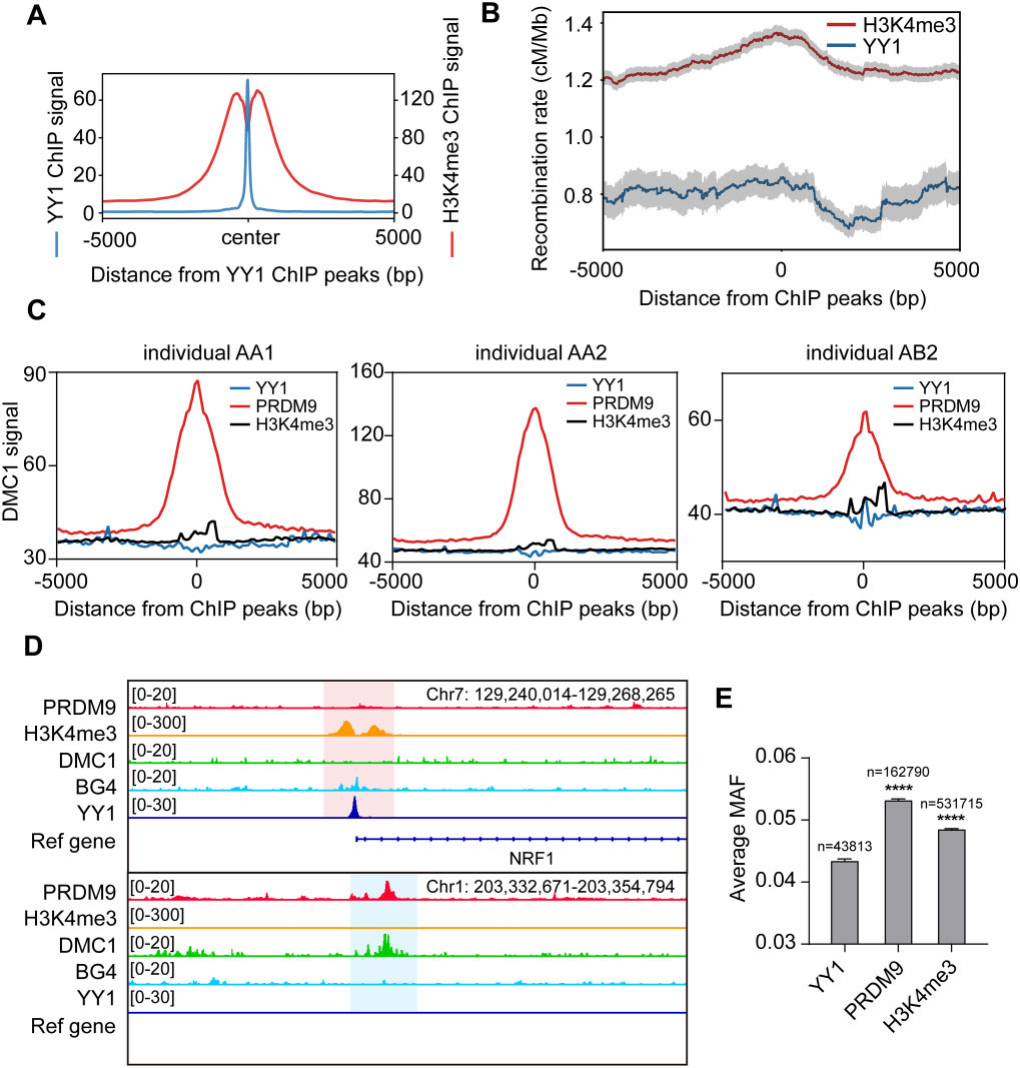
YY1 binds to nucleosome-depleted region with lower minor allele frequency. (**A**) Aggregation plot of YY1 ChIP-seq peaks for mean H3K4me3 signal and YY1 ChIP-seq signal in K562. (**B**) Average recombination rates around YY1 ChIP peaks and H3K4me3 ChIP peaks in K562. Gray shading, S.E.M. (**C**) Aggregation plot of YY1 ChIP-seq peaks, PRDM9 ChIP-seq peaks and H3K4me3 ChIP-seq peaks for DMC1 ChIP-seq signal from three different individuals. (**D**) A comparison of ChIP-seq data of PRDM9, H3K4me3, DMC1, BG4 and YY1. The pink shaded area represents the overlap of YY1, BG4 and H3K4me3 binding sites. The blue shaded area represents the overlap of PRDM9 and DMC1 binding sites. (**E**) Average minor allele frequency (MAF) of YY1, PRDM9 and H3K4me3. *P* values were calculated by using two-tailed Student's *t*-test: ****P < 0.0001. The data represent mean ± S.E.M. of results (*n* > 40 000).

To validate the role of YY1 in HR, we investigated its chromatin occupancy in conjunction with key HR-related factors, particularly PRDM9. PRDM9, a SET domain-containing protein, plays a crucial role in initiating DNA double-strand breaks and recombination by trimethylating lysine 4 in histone H3 (H3K4me3) at specific sequences. These sequences are associated with recombination hotspots, where H3K4me3 acts as a marker (6,7). Since YY1 was found to bind with the promoter regions of genes that are also enriched in H3K4me3 (58), we analyzed H3K4me3 ChIP-seq signals at YY1-binding sites and found a symmetrically enriched pattern with a dip at the center of YY1 peak, likely reflecting nucleosome depletion at YY1-binding sites (Figure 4A and Supplementary Figure S8A). Comparing recombination rates, we found that YY1-occupied regions exhibited significantly lower rates than regions marked by H3K4me3 (Figure 4B and Supplementary Figure S8B). To ascertain the impact of G4 formation on H3K4me3, we conducted H3K4me3 ChIP-qPCR at both a control region and G4-enriched genomic sites. Results revealed no significant H3K4me3 signal changes after knockdown of BLM, indicating that H3K4me3 levels remain unaffected by G4 formation (Figure 3A and Supplementary Figure S8C).

PRDM9 binds to specific sequences in the genome and recruits the meiotic recombination protein SPO11 to introduce double-strand breaks. These breaks serve as recombination sites and, with the assistance of meiotic recombination protein DMC1, a meiotic-specific recombinase expressed primarily in testis and embryonic ovary, facilitate strand invasion of the homologous chromatid (59–61). DMC1 mapping was used for identifying recombination hotspots in humans (60). Hence, we investigated the DMC1 signals on YY1, PRDM9 and H3K4me3 ChIP-seq peaks. Here, we used DMC1 ChIP-seq data generated from testis tissues from three individual human males (61). Comparing the DMC1 ChIP-seq, we observed diminished signals on YY1-occupied regions relative to PRDM9- and H3K4me3-occupied regions (Figure 4C-D and Supplementary Figure S9). However, PRDM9 peaks overlapped with DMC1 peaks, suggesting higher HR frequency at PRDM9 sites (Figure 4D and Supplementary Figure S9). Analyzing overlap between BG4, YY1, PRDM9 and DMC1 peaks revealed minimal overlap between YY1 or BG4 with PRDM9 and DMC1 (Supplementary Figure S8D). Additionally, over 40% of BG4 peaks overlapped with introns, with those overlapping YY1 peaks showing lower recombination rates (S8G). We speculated that YY1 shields G4-enriched genes from HR, distinguishing them from recombination hotspots occupied by PRDM9.

A single nucleotide polymorphism (SNP) refers to a genetic variant at a single base position in the DNA. SNPs are not evenly distributed throughout the human genome and are widely used for estimating genome recombination, showing a strong positive correlation with HR hotspots (62–64). To further support the idea of YY1’s suppression of HR, we investigated whether YY1 occupancy across the genome influences SNPs compared to PRDM9 and H3K4me3. Minor allele frequency (MAF) is the frequency of the less common allele of SNPs occurred in a given population. Those MAF variants that occur only once drive numerous selections (65). Here, we calculated the average MAF of common SNPs of YY1-, PRDM9- and H3K4me3-binding regions using the dbSNP database (65) (Figure 4E). Our analysis revealed that the average MAFs of PRDM9 and H3K4me3 binding sites were higher than those of YY1. Notably, the average MAF of PRDM9 peaks was >5%, suggesting that the variations in the PRDM9-binding sites are more common (66). These results suggest a role of YY1 in suppressing HR and are consistent with the notion that PRDM9 serves as a key determinant for the formation of recombination hotspots.

**Figure 5.**
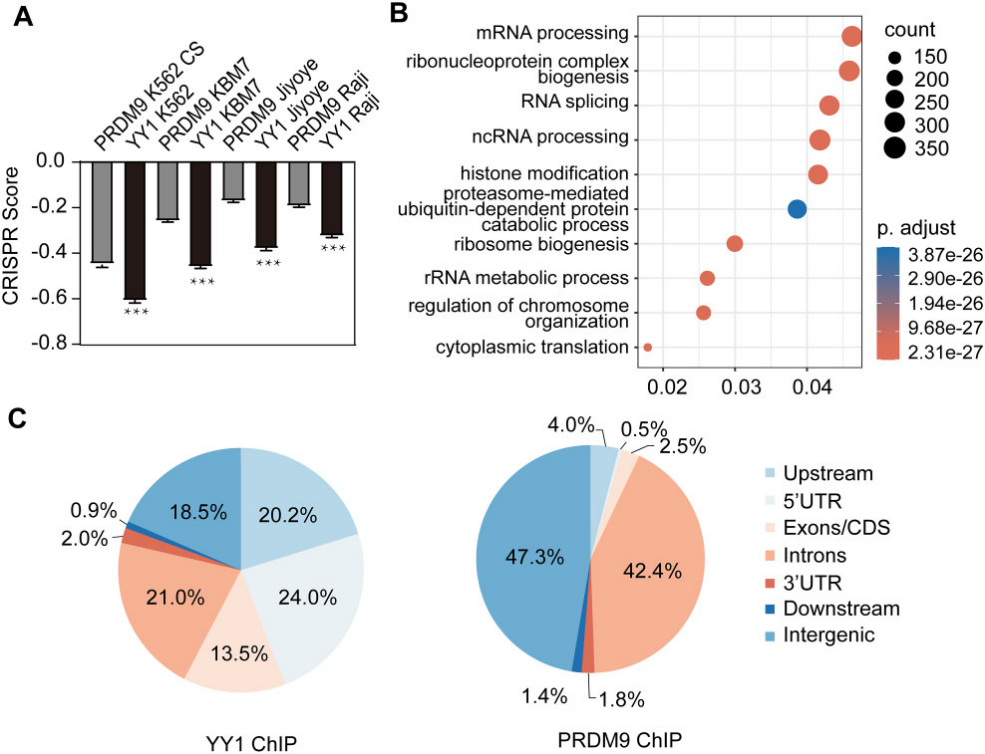
YY1 binds to essential genes to protect them from HR. (**A**) Average CRISPR scores for genes occupied by YY1 or PRDM9. (**B**) GO analysis of genes occupied by YY1. (**C**) Genomic distributions of YY1 ChIP-seq peaks and PRDM9 ChIP-seq peaks. The data in A represent mean ± S.E.M. of results.

We delved into uncovering specific patterns underlying the functions of YY1 and PRDM9. The CRISPR score is a quantitative metric used to evaluate the impact of genetic perturbations on cell fitness or phenotype. It was developed through systematic gene knockout experiments, where each gene is individually targeted for inactivation using CRISPR technology. By observing the resulting changes in cell fitness caused by gene inactivation, the CRISPR score provides insight into the functional significance of specific genes (40). CRISPR score is defined as the average log_2_ fold change in the abundance of all sgRNAs targeting a given gene after CRISPR screening. Generally, lower CRISPR score indicates a stronger effect of genetic perturbation on cell fitness or phenotype (40). CRISPR score was initially devised in KBM7, a near-haploid chronic myelogenous leukemia (CML) cell line, as the unusual karyotype allows for independent genetic screening, and was verified by another CML cell line (K562) and two Burkitt's lymphoma cell lines (Raji and Jiyoye) (40). In essence, the CRISPR score serves as a valuable tool for ranking genes based on their functional importance in cellular processes. We analyzed the CRISPR score of genes occupied by YY1 or PRDM9, and found that YY1-associated genes displayed notably lower CRISPR score than PRDM9-associated genes (Figure 5A). This indicates that YY1-modulated genes are more crucial for cell fitness and phenotype. Additionally, we performed a Gene Ontology (GO) analysis of YY1-bound genes (Figure 5B), revealing their involvement in vital biological processes such as mRNA processing, ribonucleoprotein complex biogenesis, RNA splicing, and ncRNA processing. Moreover, comparing the genomic distributions of YY1 and PRDM9 ChIP-seq peaks, we noted distinct preferences. YY1 predominantly binds to promoters (20.2%), 5′-UTRs (24.0%), and CDS (13.5%), while PRDM9 primarily targets introns (42.4%) and intergenic (47.3%) regions (Figure 5C). Together, these results collectively support the notion that YY1 binds to essential genomic regions to suppress HR, whereas PRDM9 binds to less essential regions to initiate HR. Since PRDM9-mediated HR constitutes recombination hotspots and increases genome diversity throughout evolution, we infer that YY1 may be a key determinant for cold spot maintenance and conservative gene protection (67).

## Discussion

YY1 is a transcription factor widely expressed in human cells and it assumes multiple functions in gene expression regulation and cancer progression (24). It is considered a potential prognostic marker and therapeutic target (24). In this study, we screened a group of G4-binding proteins involved in DNA metabolism and damage repair, and we identified a novel function of YY1 in suppressing HR at the genome-wide scale. Previous studies showed that aberrant G4 structure formation, BLM deficiency, and PRDM9 occupancy stimulate HR hotspots. However, it remains unclear how cells avoid undesirable HR in certain genomic regions, or what the molecular determinants are for modulating HR cold spots. Here, we found that YY1 knockdown exacerbates HR elicited by BLM depletion in cells, though YY1 knockdown alone does not appreciably affect HR (Figure 1), suggesting the presence of other G4-binding proteins in suppressing HR at G4 structure sites. Moreover, treatment of cells with G4-binding small molecules, PDS and TMPyP4, which impede YY1 and BLM from binding to G4 structures, thereby resulting in significant elevations in HR (Figure 2C and D). Therefore, we propose that G4 structures in the genome serve as anchors for the initiation of HR, which can be suppressed by BLM or YY1 via distinct mechanisms, i.e. through unwinding and interacting with G4 structures, respectively.

We also found that genomic regions with higher YY1 occupancy exhibit lower recombination rates in the human population (Figure 2A, B and Supplementary Figure S5A–S5E). The SCE assay monitors HR in mitosis, whereas SNP maps from the 1000 Genomes Project mainly reflect HR in meiosis (26,36). Given the high conservation of YY1 among vertebrate species, we hypothesize that YY1 binding to G4 structures may represent a common mechanism for inhibiting HR during both mitosis and meiosis across vertebrates, not just in humans. This hypothesis warrants further investigation to elucidate its validity in other vertebrate species. Moreover, we showed that YY1 suppresses HR by impairing the chromatin localization of RAD51, a recombinase active at DNA double-strand breaks (Figure 3B, C).

In this study, we did not determine the frequency of SCEs at the single-gene level. As shown by the SCE assay images and quantification (Figure 1B, C, Figure S3), each chromosome exhibited fewer than one SCE event among over 100 chromosomes analyzed. Given that chromosome lengths range from 59 to 250 Mb, SCEs are rare events in normal cells. Additionally, while the resolution for SCE detection has improved and can reach up to 100 kb with recent sequencing techniques, this is still insufficient for pinpointing single genes (23). In the future, employing more precise techniques to analyze SCE events at the single-gene level will better support our inferences. PRDM9 was identified as a key determinant for initiating HR (6,7). Here, we found that YY1 suppresses HR as its binding sites exhibit lower recombination rate and diminished average MAF of SNPs (Figure 4), and genes associated with YY1 display significantly lower CRISPR scores than those associated with PRDM9 and involved in regulating essential biological processes (Figure 5A and B). Moreover, PRDM9 is preferentially localized at introns and intergenic regions, whereas YY1 binds to promoters, 5′-UTRs, and CDS of protein-coding genes (Figure 5C). These findings support the notion that YY1 protects essential regions of the genome from HR, whereas PRDM9 initiates HR in non-essential regions to promote genetic diversity during evolution.

In conclusion, our results showed that YY1 suppresses HR partly by binding to G4 structures, and contributes to the conservation of crucial genes in biological processes in human cells. Considering the physiological importance of YY1 and its conservation across multiple mammalian species (68), it will be important to develop therapeutic approaches targeting specifically the YY1-G4 interaction.

## Supplementary Material

gkae502_Supplemental_File

## Data Availability

The CUT&Tag data generated in this study have been deposited into the NCBI GEO database with the accession number GSE254872. The ChIP-seq data for bioinformatic analysis in Figure 1A were obtained from NCBI GEO database with accession numbers presented in Supplementary Table S4. The HEK293T YY1 ChIP–seq data were obtained from NCBI GEO database with the accession number of GSE128106; The YY1 ChIP-seq data for Ishikawa, SK-N-SH, HepG2, HCT116 and K562 cell lines were obtained from NCBI GEO database with the accession numbers of GSM1010753, GSM1010897, GSM803381, GSM803354 and GSM935368. The BG4 ChIP-seq data was obtained from NCBI GEO database with the accession numbers of GSE107690. The G4access data was obtained from NCBI GEO database with the accession numbers of GSE187007. The DMC1 and PRDM9 ChIP-seq data were obtained from NCBI GEO database with the accession numbers of GSE59836 and GSE99407, respectively. The H3K4me3 ChIP-seq data for K562, HepG2 and HCT116 were obtained from ENCODE database with the accession numbers of ENCFF507QPR, ENCFF962DDH and ENCFF534WDD. The RAD51 ChIP-seq data was obtained from NCBI GEO database with the accession numbers of GSE91838. The dbSNP data was obtained from NCBI dbSNP database with version of human 9606 b151 GRCh37p13 common vcf file.

## Code availability

Custom codes used in this work are available from https://github.com/Margooooovo/YY1_HR_code and https://doi.org/10.5281/zenodo.11368863.
