# Supplementary material for: YY1 is involved in homologous recombination inhibition at guanine quadruplex sites in human cells
Source: Nucleic Acids Res. 2024 Jun 13;52(13):7401–13. doi: 10.1093/nar/gkae502 (PMC11260479; doi:10.1093/nar/gkae502)
Supplement: gkae502_Supplemental_File [file gkae502_supplemental_file.pdf]

YY1 is involved in homologous recombination inhibition at guanine quadruplex sites in human cells

Xinyu Cui[1,2], Chengwen Zhang[1,2], Chunqing Fu[1,2], Jinglei Hu[1,2], Tengjiao Li[1,2], and Lin Li[1,2]*

1. Shanghai Frontiers Science Center of Drug Target Identification and Delivery, School of Pharmaceutical Sciences, Shanghai Jiao Tong University, Shanghai 200240, China.

2. National Key Laboratory of Innovative Immunotherapy, Shanghai Jiao Tong University, Shanghai 200240, China.

*Corresponding Author: Lin Li, E-mail: Linli.sjtu@sjtu.edu.cn

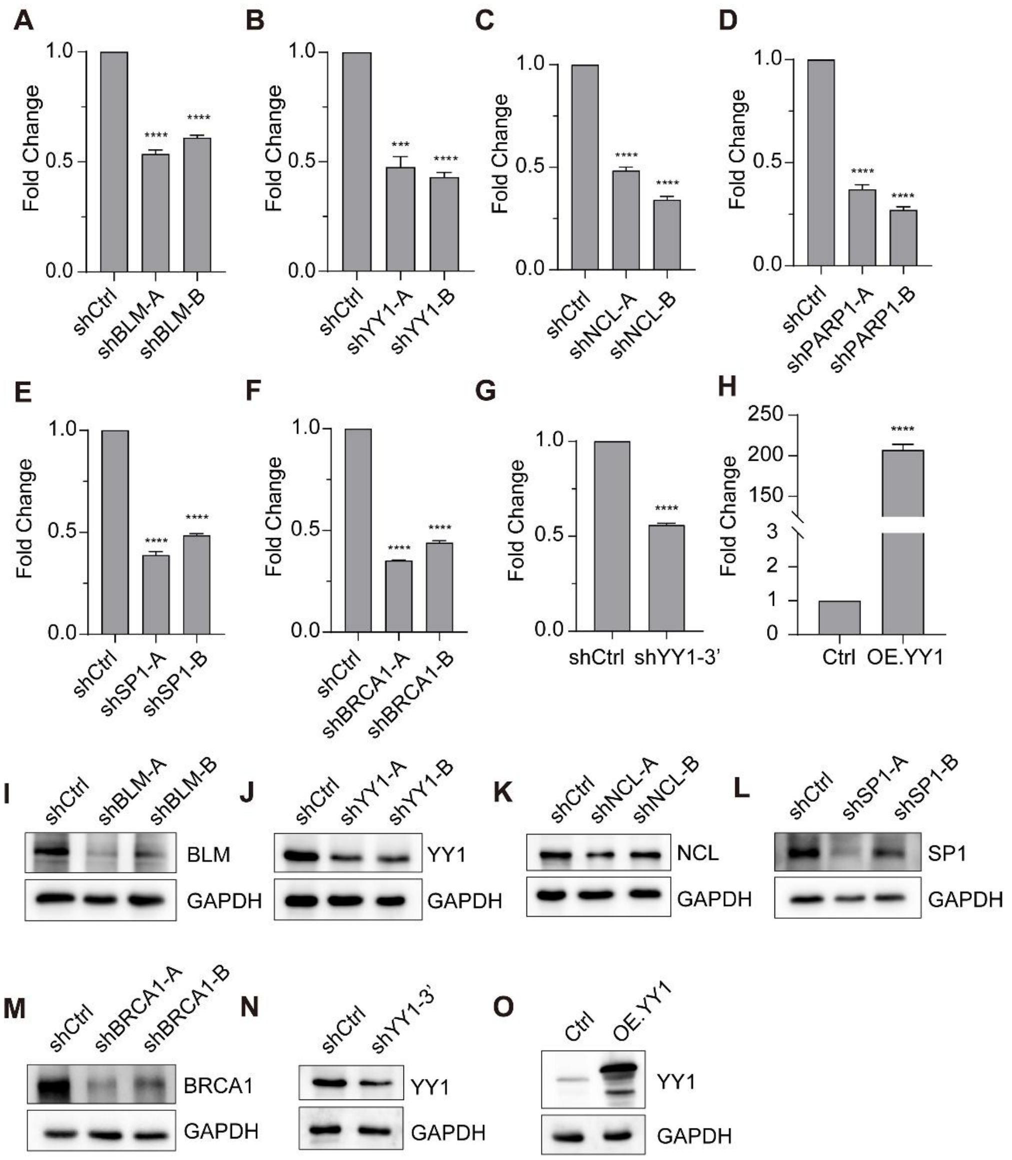

Supplementary Figure 1. Confirmation of knockdown and overexpression efficiency in SCE assay. (A-G) Knockdown efficiency of shRNA used in SCE assay on RNA level. (H) Overexpression efficiency in SCE rescue experiment on RNA level. (I-N) Knockdown efficiency of shRNA used in SCE assay on protein level. (O) Overexpression efficiency in SCE rescue experiment on protein level. The P values were calculated by using two-tailed Student's t-test: ***P < 0.001; ****P < 0.0001. The data represent mean ± S.E.M. of results (n = 3 in A-O).

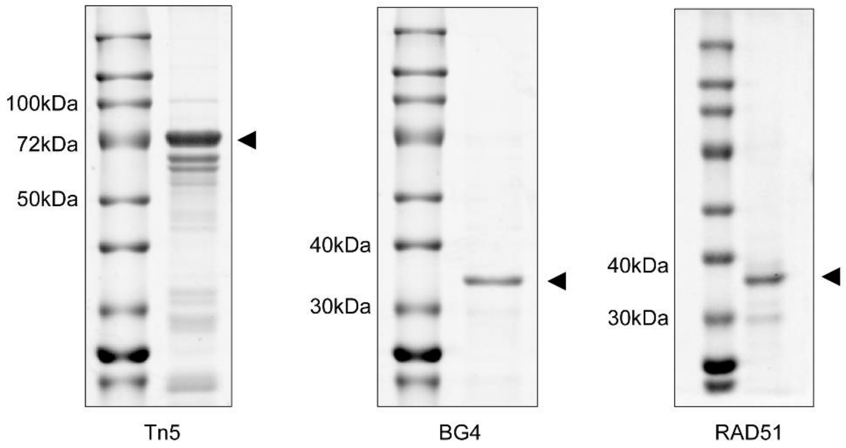

Supplementary Figure 2. Coomassie blue staining of purified 10 μL 10 μM Tn5 (Left), 10 μL 2 μM BG4 (Middle), and 10 μL 5 μM RAD51 (Right).

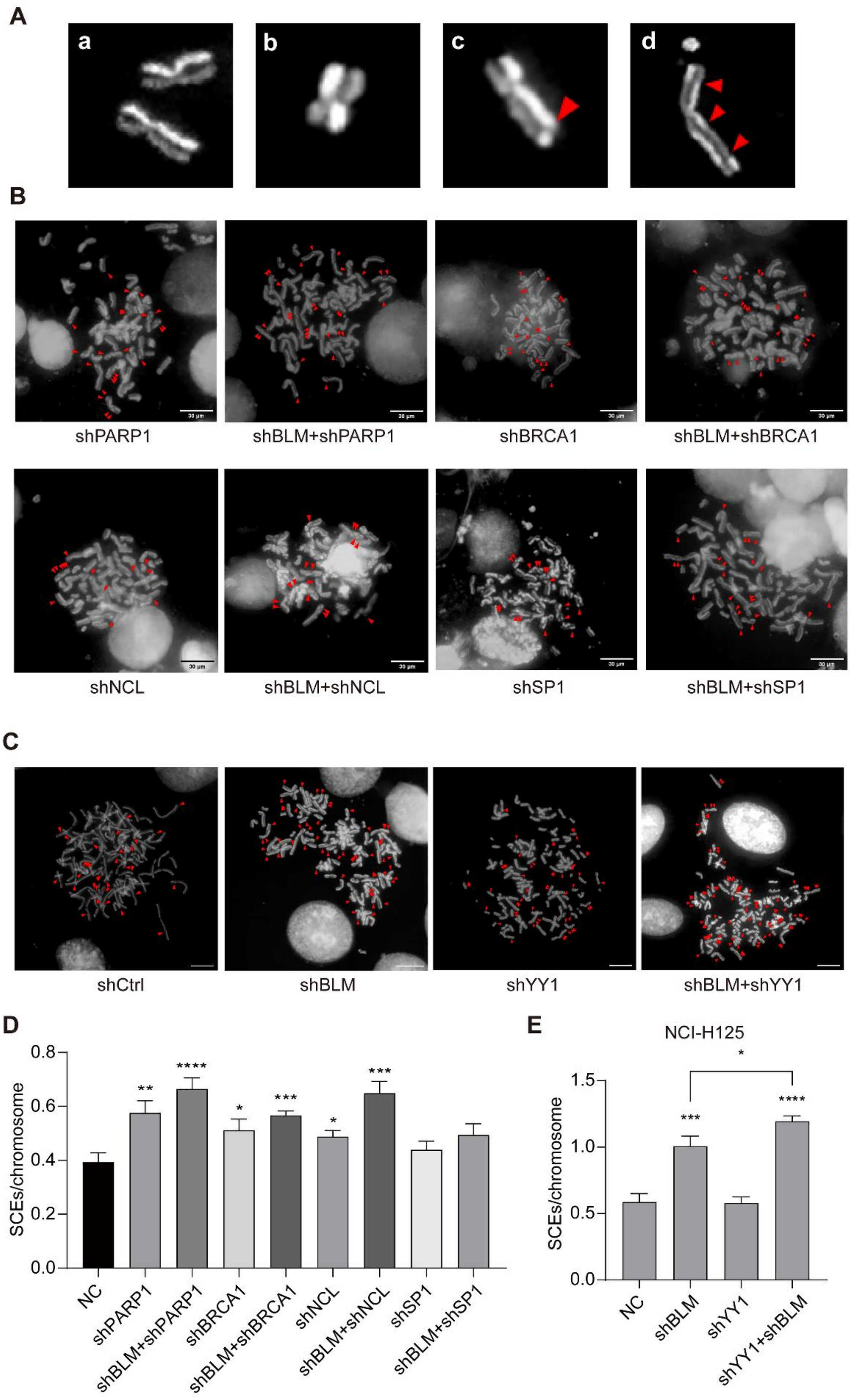

Supplementary Figure 3. SCE assay in HEK293T and NCI-H125.

(A) Demonstrations of sister chromatid exchange events (SCEs). a: no SCE; b: no SCE and the exchange of color was caused by the twist of the centromere; c-d: SCEs happened at sites marked

with red arrowhead. (B) SCEs were shown in HEK293T cells after knockdown of BLM, G4-binding proteins, alone or in combination. SCEs were marked with red arrowhead. (C) SCEs were shown in NCI-H125 cells after knockdown of BLM, YY1, alone or in combination. SCEs were marked with red arrowhead. (D) Quantification of SCEs per chromosome after shRNA-mediated knockdown of BLM and the indicated G4-binding proteins, alone or in combination in HEK293T. (E) Quantification of SCEs per chromosome after knockdown of BLM, YY1, alone or in combination in NCI-H125. P values were calculated by using two-tailed Student's t-test: $^{*}P < 0.05$; $^{**}P < 0.01$;$^{***}P < 0.001$; $^{****}P < 0.0001$. The data represent mean ± S.E.M. of results (n > 150 in A and C, n > 600 in B and D).

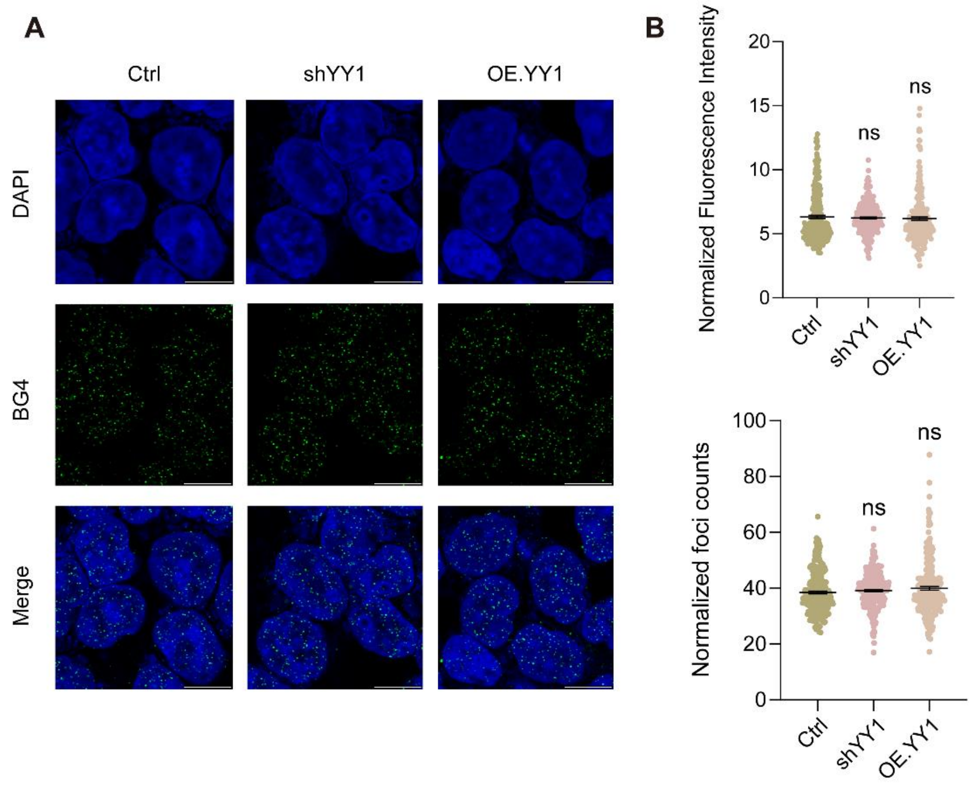

Supplementary Figure 4. (A) Representative fields of view showing G4 foci formation detected by immunofluorescence in control (Ctrl), shYY1 and OE.YY1 HEK293T cells. Nuclear staining (blue), BG4 (green) and the merged channels are reported. Scale bars = 10 µm. (B) Quantification of BG4 nuclear staining detected by immunofluorescence in control (Ctrl), shYY1 and OE.YY1 HEK293T cells. BG4 integrated fluorescence intensity within nuclei was normalized by the corresponding nuclear area. P values were calculated by using two-tailed Student's t-test: ns: $P > 0.05$. The data represent mean ± S.E.M. of results ($n > 200$).

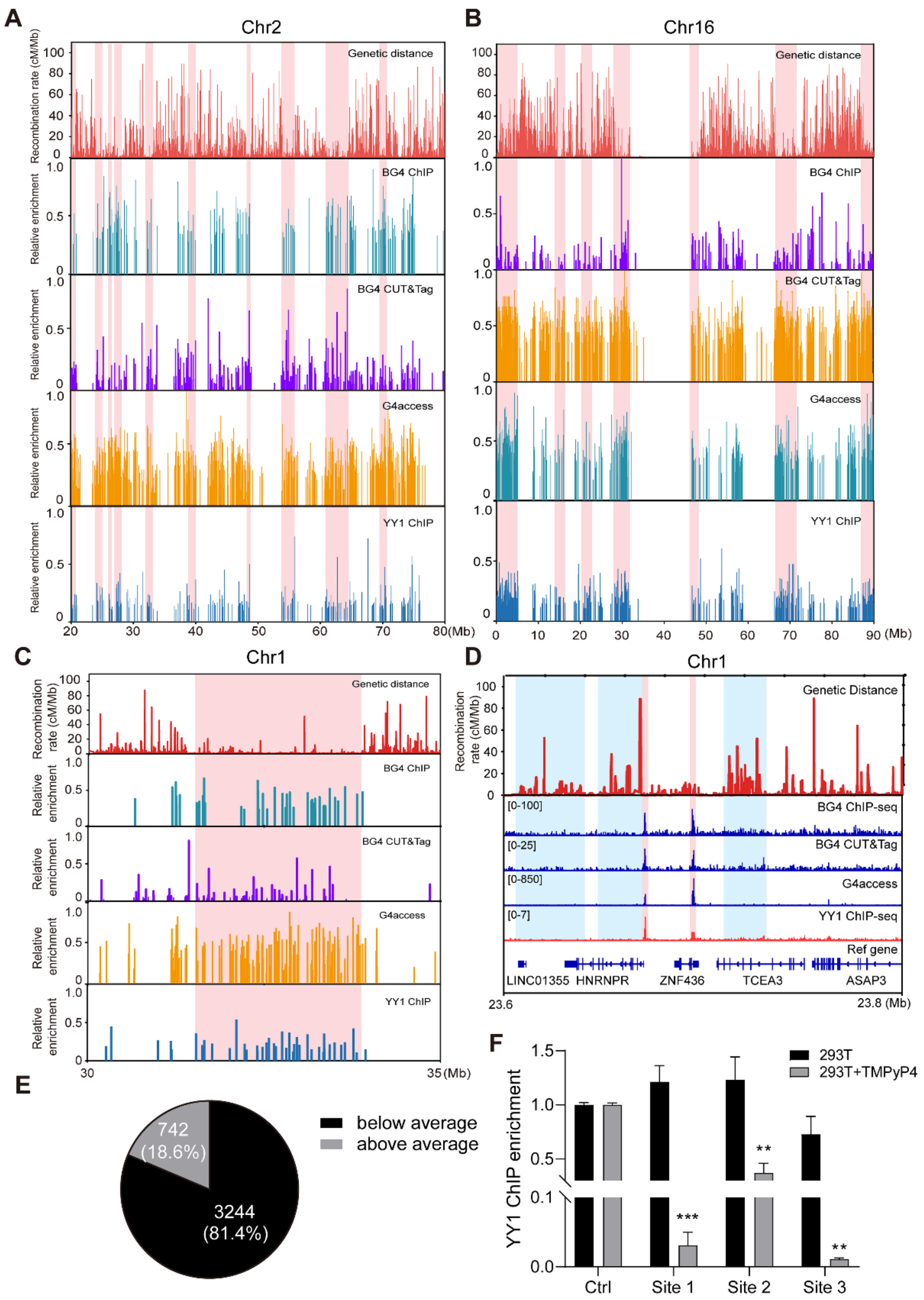

Supplementary Figure 5. (A-B) A comparison of genetic map and ChIP-seq peaks of BG4 and YY1 in regions on (A) chromosome 7 (60 Mb-150 Mb) and (B) chromosome 16 (0-90 Mb). Five datasets were used, including BG4 ChIP-seq (second panel), BG4 CUT&Tag (third panel), G4access (fourth panel) and YY1 ChIP-seq (bottom panel). Regions with low recombination rates and high sequencing enrichment are marked with pink shaded areas. (C) A zoomed in region of 30-35 Mb of Figure 2B. Regions with low recombination rates but high ChIP-seq enrichment were marked in pink columns. (D) A further zoomed region on Chr1 (23.6 Mb-23.8 Mb). Regions with low recombination rates but high ChIP-seq enrichment were marked in pink columns, and with high

recombination rates but low ChIP-seq enrichment were marked in blue columns. (E) Percentage of the recombination rates of G4 peaks overlapping with YY1 below or above average.(F) YY1 ChIP-qPCR enrichment in control HEK293T cells and TMPyP4 treated HEK293T cells in control region and G4 sites 1-3 ($n = 3$). The P values were calculated by using two-tailed Student's t-test: $^{**}P < 0.01$; $^{***}P < 0.001$. The data represent mean ± S.E.M. of results.

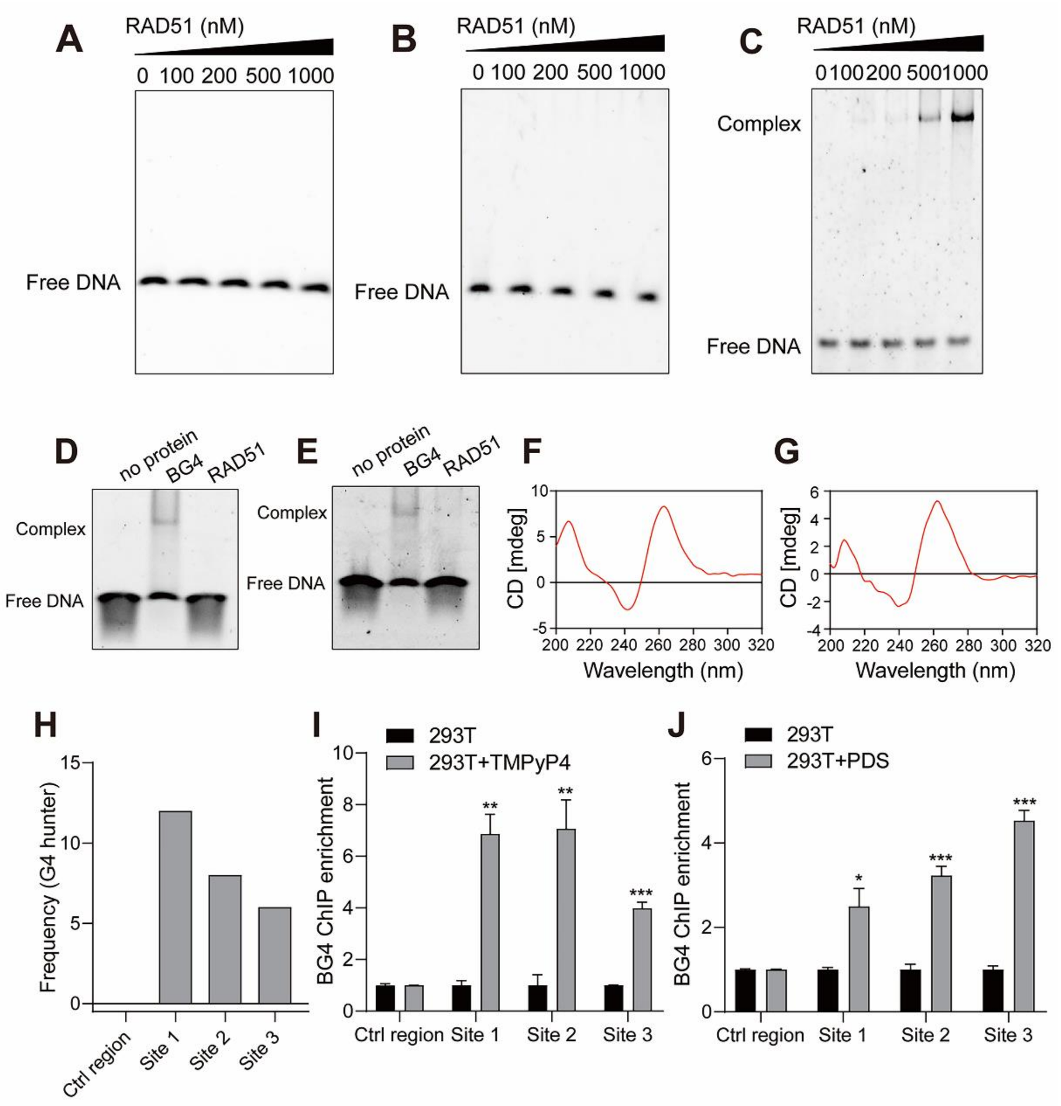

Supplementary Figure 6. (A-B) EMSA results showing the binding of RAD51 toward G4 structures (c-KIT in A and c-MYC in B). (C) EMSA results showing the binding of RAD51 toward ss-DNA. (D-E) EMSA results showing the binding of 1000 nM RAD51 toward G4 structures comparing to 800 nM BG4 (c-KIT in D and c-MYC in E). (F-G) CD spectra of c-KIT G4 (F) and c-MYC G4 (G). (H) Frequency (quadruplexes found per kilobase) of predicted G4 of control region and G4 sites 1-3 by G4 hunter. (I-J) BG4 ChIP-qPCR enrichment in control HEK293T cells and TMPyP4 (I) or PDS (J) treated HEK293T cells in control region and G4 sites 1-3 (n = 3). The P values were calculated by using two-tailed Student's t-test: $*P < 0.05$; $**P < 0.01$; $***P < 0.001$. The data represent mean ± S.E.M. of results.

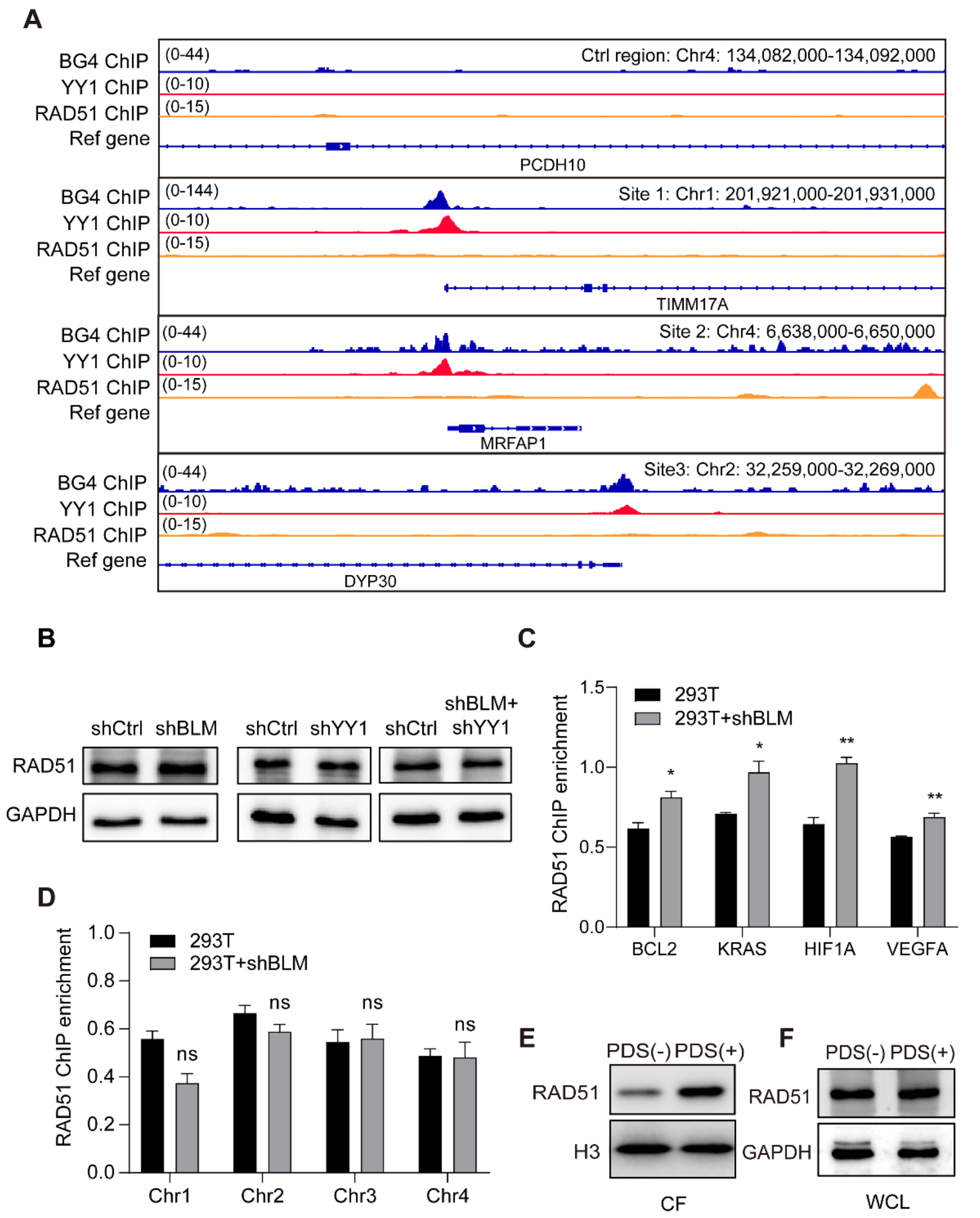

Supplementary Figure 7. (A) ChIP-seq signals of BG4, YY1 and RAD51 in control region (first panel) and G4 sites 1-3 used in ChIP-qPCR (panel 2-4). (B) Western blotting anti-RAD51 and anti-GAPDH results in shCtrl, shBLM, shYY1 and shBLM+shYY1 HEK293T cells using whole cell lysate. (C) RAD51 ChIP-qPCR enrichment in 4 well-established G4-rich gene regions in HEK293T cells and BLM knockdown HEK293T cells (n=3). (D) ChIP-qPCR enrichment of RAD51 in non-G-rich but high recombination rate regions of Chr1-Chr4 (n=3). (E) Western blotting anti-RAD51 and anti-histone H3 results of the chromatin fraction (CF) of HEK293T cells with or without PDS treatment. (F) Western blotting anti-RAD51 and anti-GAPDH results of whole cell lysate (WCL) of HEK293T cells with or without PDS treatment. The P values were calculated by using two-tailed Student's t-test: *P < 0.05; **P < 0.01. The data represent mean ± S.E.M. of results.

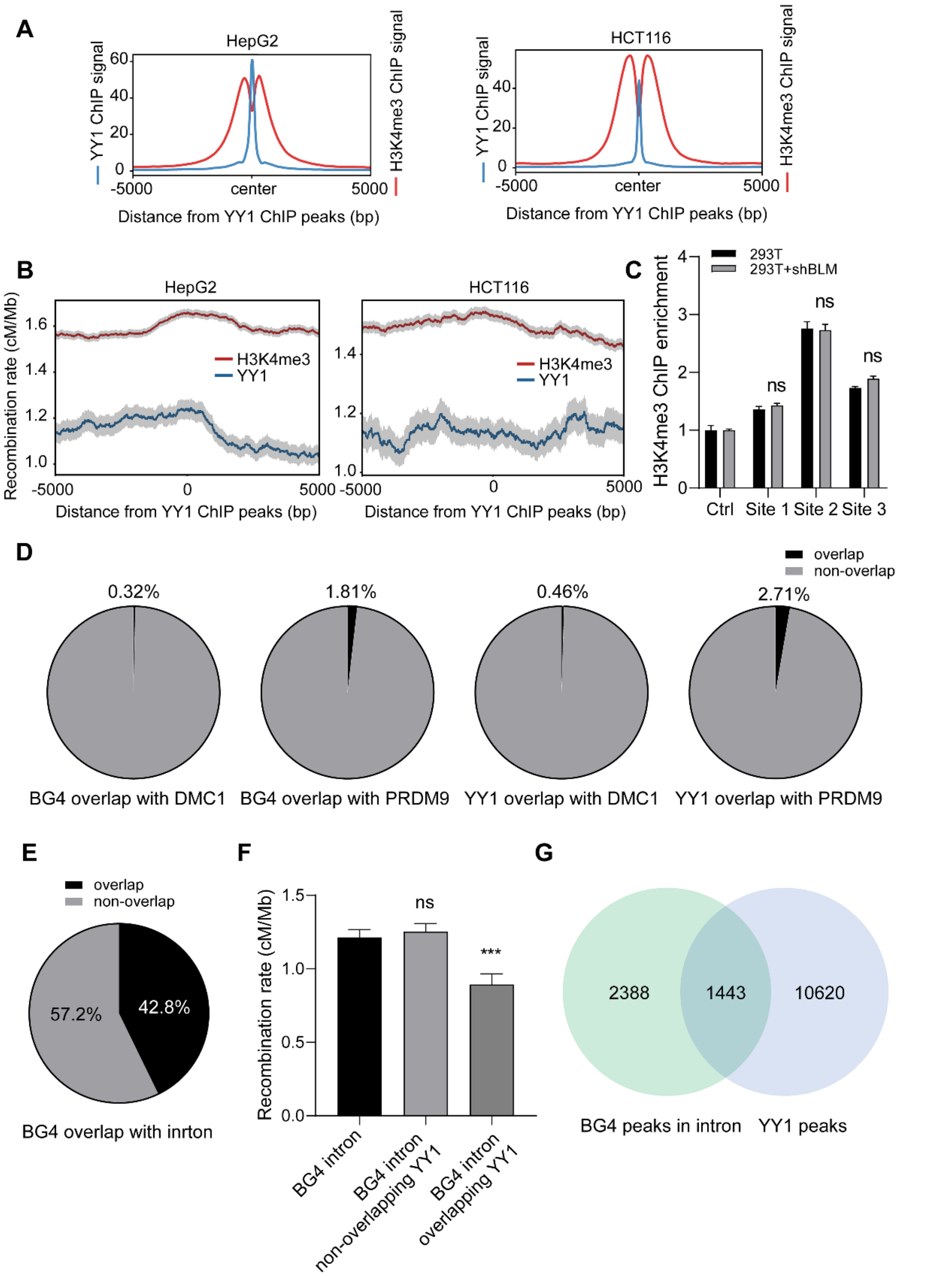

Supplementary Figure 8. (A) Aggregation plot of YY1 ChIP-seq peaks for mean H3K4me3 signal and YY1 ChIP-seq signal in three different cell lines. (B) Average recombination rates around YY1 ChIP peaks and H3K4me3 ChIP peaks in three different cell lines. Gray shading, S.E.M. (C) H3K4me3 ChIP-qPCR enrichment in HEK293T and shBLM HEK293T cells in control region and G4 sites 1-3 (n=3). Regions for qPCR were shown in Figure S5D. (D) BG4 and YY1 overlapping with DMC1 and PRDM9. (E) BG4 overlapping with introns. (F) Average recombination rate of BG4 peaks in introns, BG4 peaks in introns non-overlapping with YY1 and BG4 peaks in introns overlapping with YY1 (n > 1000). (G) Venn diagram of BG4 peaks in intron and YY1 peak. The P

values were calculated by using two-tailed Student's t-test: ***$P < 0.001$. The data represent mean ± S.E.M. of results.

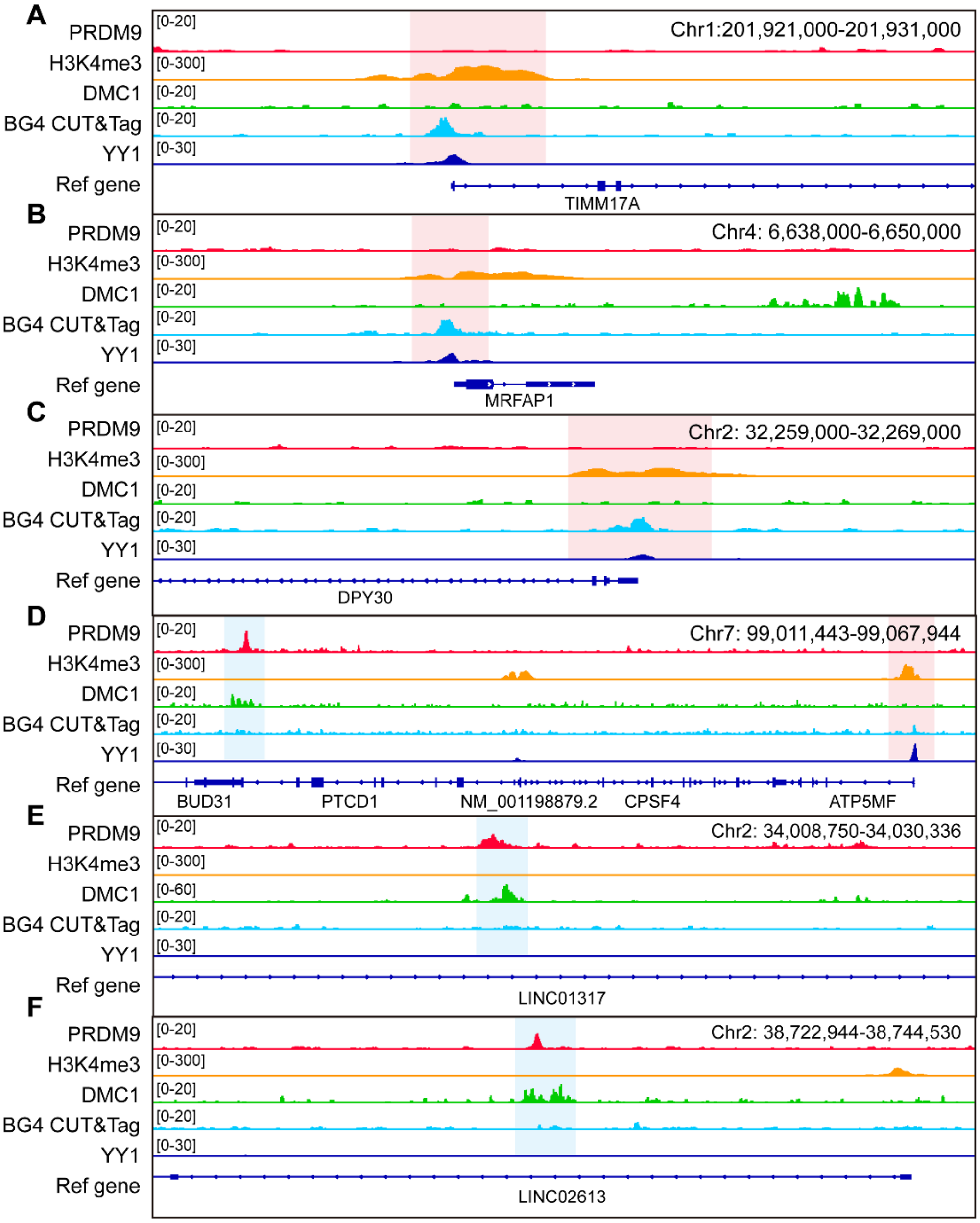

Supplementary Figure 9. (A-F) A comparisons of ChIP-seq data of PRDM9, H3K4me3, DMC1, BG4 and YY1. The pink shaded area represents the overlap of YY1, BG4 and H3K4me3 binding sites. The blue shaded area represents the overlap of PRDM9 and DMC1 binding sites. A-C were G4-enriched regions used in ChIP-qPCR, D-F were three random regions.

Supplementary Table 1. Target sequences of shRNA.

Gene	Target sequence (5’-3’)
NC (Ctrl)	CAACAAGATGAAGAGCACCAA
BLM-A	ACCGAATCTCAATGTACATAG
BLM-B	GACGCTAGACAGATAAGTTTA
YY1-A	GACGACGACTACATTGAACAA
YY1-B	CCTCCTGATTATTCAGAATAT
PARP1-A	GCTTCACATATCAGCAGGTTA
PARP1-B	CGACCTGATCTGGAACATCAA
NCL-A	GCACTTGGAGTGGTGAATCAA
NCL-B	CGGTGAAATTGATGGAAATAA
SP1-A	GCTGGTGGTGATGGAATACAT
SP1-B	CCACTCCTTCAGCCCTTATTA
BRCA1-A	AGAATCCTAGAGATACTGAA
BRCA1-B	GAGTATGCAAACAGCTATAAT

Supplementary Table 2. Primers for ChIP-qPCR.

Primer name	Primer sequence (5'-3')	Region
ChIP-Ctrl-F	CCAGTTCACACGGCTCAAAA	chr4:13408200
ChIP-Ctrl-R	AGTCGTATGAAGAACTAAGAAGAGC	0-134092000
ChIP-Site1-F	GCGCGTACTCCTCCATCTT	chr1:20192100
ChIP-Site1-R	TGCAGCGGGAAATGGTGAAC	0-201931000
ChIP-Site2-F	GCAGAGTAACGGCGAACAAA	chr4:6638000-
ChIP-Site2-R	GCCTCTCTTTCTAGACGTGC	6650000
ChIP-Site3-F	GGACTGACGATGGTTCTGC	chr2:32259000-
ChIP-Site3-R	TTCGTTCTTAAGAGCCCTGCAC	32269000
Chr1-F	TTCCAACCCTTCCCCATTCC	chr1:10204002
Chr1-R	TTGCTTTCCAGACACAGAATAGGA	9-102040534
Chr2-F	CTCACCTGCCTTTGATAGACTT	chr2:13941441
Chr2-R	GCAACAGAATTTTGCACAGTCAT	8-139415435
Chr3-F	TAACACGCTCTTTGCCACTCT	chr3:17566953
Chr3-R	CAGGGAGGCGAACAATTCTCT	3-175670278
Chr4-F	CCTGTGCTACATAAACTACTCTGGA	chr4:33220693-
Chr4-R	CGTCTCCATTCCCCACCATT	33221757
KRAS-F	TCCGAGCACACCGATGAGT	
KRAS-R	TGAAGAAGAATCGAGCGCGG	
BCL2-F	CATTCTCTGCACAGCCCGAC	
BCL2-R	TCAGAGGAGGGCTCTTTCTTT	

VEGFA-F	TCTGGACAGAGTTTCCGGGG
VEGFA-R	CCGCAATGAAGGGGAAGCTC
HIF1A-F	CTCTTTCCTCCGCCGCTAA
HIF1A-R	GCCGCTCTCAGCCAATCAG

Supplementary Table 3. Sequences of probes used in EMSA.

Probe	Sequence (5'-3')
c-KIT	AGGGAGGGCGCTGGGAGGAGGG (TAMRA)
c-MYC	TGAGGGTGGGGAGGGTGGGGA (TAMRA)
ss-DNA	ATGGTGTGTGTAGGTTAATGTGAGGAGGAGAGGTGAAGAAGG AGGAGAGAAGAAGGAGGC

Supplementary Table 4. Cell lines and corresponding accession numbers used in Figure 1A.

	G4-binding protein	Cell line	Accession number
a	YY1	HEK293T	GSE128106
b		ECC-1	GSM1010753
c		SK-N-SH	GSM1010897
d		SK-N-SH	GSM1010897
e	PARP1	MDA-MB-231	GSM6443233
f		HEK293T	GSE74954
g	SP1	HepG2	GSM2423332
h		HEK293T	GSE105814
i		HCT-116	GSM1010902
j		K562	GSE92217
k		MCF-7	GSE92014
l	BRCA1	HelaS3	GSM935552
m		HepG2	GSM935609
n		K562	GSE105342
o	DNMT1	K562	GSM2424239
p		K562	GSE92213
q	ATRX	HepG2	GSM5215280
r	MAZ	HepG2	GSE169764

s		A549	GSE91939
t		HelaS3	GSM935272
u		IMR90	GSM1003613
v		K562	GSM935337
w		MCF-7	GSE91633

Supplementary Table 5. Regions of G4 sites 1-3 and control region used for G4 hunter analysis.

Site	Region (homo sapiens, hg19)	Sequence (5'-3')
Control	chr4: 134086200-134086699	CATATATTCATATATATACACATATATATATACATACATATAT TCATATATATATGAATATACATATGTTGGAATGAGTTAGTT TTTAATTGTAGTTTTTTTCCCTAATCTGTTTTAGCCAGTT CACACGGCTCAAAATTTAGGGTTTGGGTTGACGTGTGA CCTGTGGTGGAAAATTTGCAGAAGAATAGCTATTTTGTT GTTTTCACTTCTGCTCTTCTTAGTTCTTCATACGACTTTA ATCATGTCTAGACTGGTTTTTTTTTTTAATTTAACTTTTA GTATTTAATGTCATCTTAGAGTCATCCAATTATATTTAAA ATTACACCATGACTCTGACTTAATAATCATAAAAAATTTA TAGTTAGTCTGATAATACAAACTCTAAAACAAAACTATT CCTGACCATTTTGCCCCTCAAATTCATATGGTGATAGTTT TTGATACTTGTGTCCTTTTTCATTCAGAATAGAAATAGTG TCATCTTCTAAGTCTAAAGT
Site 1	chr1: 201924300-201924799	GGGGAAGGGAAGGCGGAAGGGAAGGGAAAGAAAGAA GCGTATTTAGGCAGTATCGCGTTGATTTCAGTCCACGGA ACCTCTGGTGACCGGGTAGTGTGACTCCGTCATTGGTA GCCCCCCGCCCACACAGCCGGGACTCGCGGCCTTTGAG GACGTGGGCAGGGCCAGTCTCGCCACGCCGCGGCCAA TGCCCCGACCACACGCTGGCCACAGGAGTCCTGGCTCC CATTGGCTGCAGCGGGAAATGGTGAACCAATGCTCATA GACCTTAACGCCCTCCTCTCGGGATCACTTCCGCCTCTG GGGTCAGGCTCCGCCCAGCTTGCCCGGCATCACTCGCG GCATTGGAGTCAAGATGGAGGAGTACGCGCGAGAGCC TTGGTGAGCTTCACCGCTGTCTTTGCATTTCTCTTGCCC CCCTGCCCACTGCCCTCCTTCTCCCTTGTCCAGGCTCCC AAGGTGCAAGCCAGACTCAACCGCAGCCTCGTCGACT TGGGCCT
Site 2	chr4: 6642000-6642499	TGGTCTCGAATTCCTGACCTCAAGCCATCCACCCACCTC GCCTCCCAACGTGCTGAGATTACAGGCGTGAGCCACCG CGCCTGGGAAGTGCGGGAGGCCTTTTTAAAAAATGTAA ACTCGCGTGCTCAAGAAGCCCTATCGGAGGGAAACACC CAGAAGAGGCCACCCTCCAAACACAGGGTAAAACAGG GGTGACGCACAGAGGTAACGGGAAAGCAGGAACGAGC GAGAGCGTGCGGTCCCGTACCTGCGCGTGCCTGCGTGC GCGTCCGTGCGGCCGCGACGCCGCCGCCGGTGCCTCTC TTTCTAGACGTGCGCGCGCCCGCAGCTCGCCCCCCTGA

Site 3	chr2: 32264600-<br>32265099	CCGTAAAGAGGCCCGGCTGTGTCGTGAAAGGGGCCGC<br>AACGCGCAGAGCGCTGGTTGACGGCCGGGACTCCATTT<br>TGTTCGCCGTTACTCTGCGCGTAAGTCGCTTGTCCGTGG<br>CTTCTCTGAGAAGAAAAGTTGAAAAAGGGTAAAAGTT<br>TTCAGGAA<br>TTCAACACGAAGACTCCAGGATGGAGTGCAGCCCAGC<br>CCCCGACCCCGCCCCTCACCCCCACCTGGGCGCGGGAG<br>CACCACGAGCAGTAGAGCAGCAGAGTGGGACAGTCCA<br>CGACTGGGCGCCTACATGGGGTCTGGAAACTCTACGAC<br>AGTCCCAAGTACCTAGAATAAGTGAGAAAGTGGGGGA<br>AAGGGAGCTGATTACCCGCGCCCAAGCCGTTCGTTCTT<br>AAGAGCCCTGCACCGCGCCACCAGCTCCCAGCACAAA<br>CAGCTCCGGCCGTAAGTGACGGCTGTCGCACGACTGCC<br>GCGCCTGCGCAGAACCTCCCGGCGGACAAGAAGCTGG<br>GAGGCGCGCAGAACCATCGTCAGTCCCCGAGGCTCTCT<br>GCAGTCCGCGTGAGTCCTGGCCAAGGTCTGGCCTCGGA<br>AGCTACGAGCTACAATCTTCCTTCATCCTACCCACCCAC<br>GAAGAGCGCCACCCCAGCTGCAGACCAAGTGACCAGC<br>CCAGTTAGAAG
